# Vitamin C inactivates c-Jun N-terminal kinase to stabilize heart and neural crest derivatives expressed 1 (Hand1) in regulating placentation and maintenance of pregnancy

**DOI:** 10.1007/s00018-024-05345-6

**Published:** 2024-07-15

**Authors:** Haibin Zhu, Huan Luo, Xiaowei Wu, Hangyang Bao, Yingying Shu, Xing Ji, Xueying Fan, Yibin Pan, Chao Tang, Ximei Wu, Hongfeng Ruan

**Affiliations:** 1 Department of Gynaecology, the First Affiliated Hospital, Zhejiang Univerisity School of Medicine, Hangzhou, 310009 China; 2 Department of Pharmacy, the Second Affiliated Hospital, Zhejiang University School of Medicine, Hangzhou, 310009 China; 3 Institute of Orthopaedics and Traumatology, The First Affiliated Hospital of Zhejiang Chinese Medical University (Zhejiang Provincial Hospital of Traditional Chinese Medicine), Hangzhou, 310053 Zhejiang China; 4 Department of Pharmacology, Zhejiang Univerisity School of Medicine, Hangzhou, 310058 China; 5 Department of Obstetrics and Gynaecology, the Affiliated Sir Run Run Shaw Hospital, Zhejiang Univerisity School of Medicine, Hangzhou, 310016 China; 6 Shulan International Medical College, Zhejiang Shuren University, Hangzhou, 310015 China

**Keywords:** Vitamin C, Hand1, Placentation, JNK inactivation, Trophoblast giant cell

## Abstract

Vitamin C (VC) serves as a pivotal nutrient for anti-oxidation process, metabolic responses, and stem cell differentiation. However, its precise contribution to placenta development and gestation remains obscure. Here, we demonstrated that physiological levels of VC act to stabilize Hand1, a key bHLH transcription factor vital for the development trajectory of trophoblast giant cell (TGC) lineages, thereby promoting the differentiation of trophoblast stem cells into TGC. Specifically, VC administration inactivated c-Jun N-terminal kinase (JNK) signaling, which directly phosphorylates Hand1 at Ser48, triggering the proteasomal degradation of Hand1. Conversely, a loss-of-function mutation at Ser48 on Hand1 not only significantly diminished both intrinsic and VC-induced stabilization of Hand1 but also underscored the indispensability of this residue. Noteworthy, the insufficiency of VC led to severe defects in the differentiation of diverse TGC subtypes and the formation of labyrinth's vascular network in rodent placentas, resulting in failure of maintenance of pregnancy. Importantly, VC deficiency, lentiviral knockdown of JNK or overexpression of Hand1 mutants in trophectoderm substantially affected the differentiation of primary and secondary TGC in E8.5 mouse placentas. Thus, these findings uncover the significance of JNK inactivation and consequential stabilization of Hand1 as a hitherto uncharacterized mechanism controlling VC-mediated placentation and perhaps maintenance of pregnancy.

**Graphical Abstract:**

**Figure:**
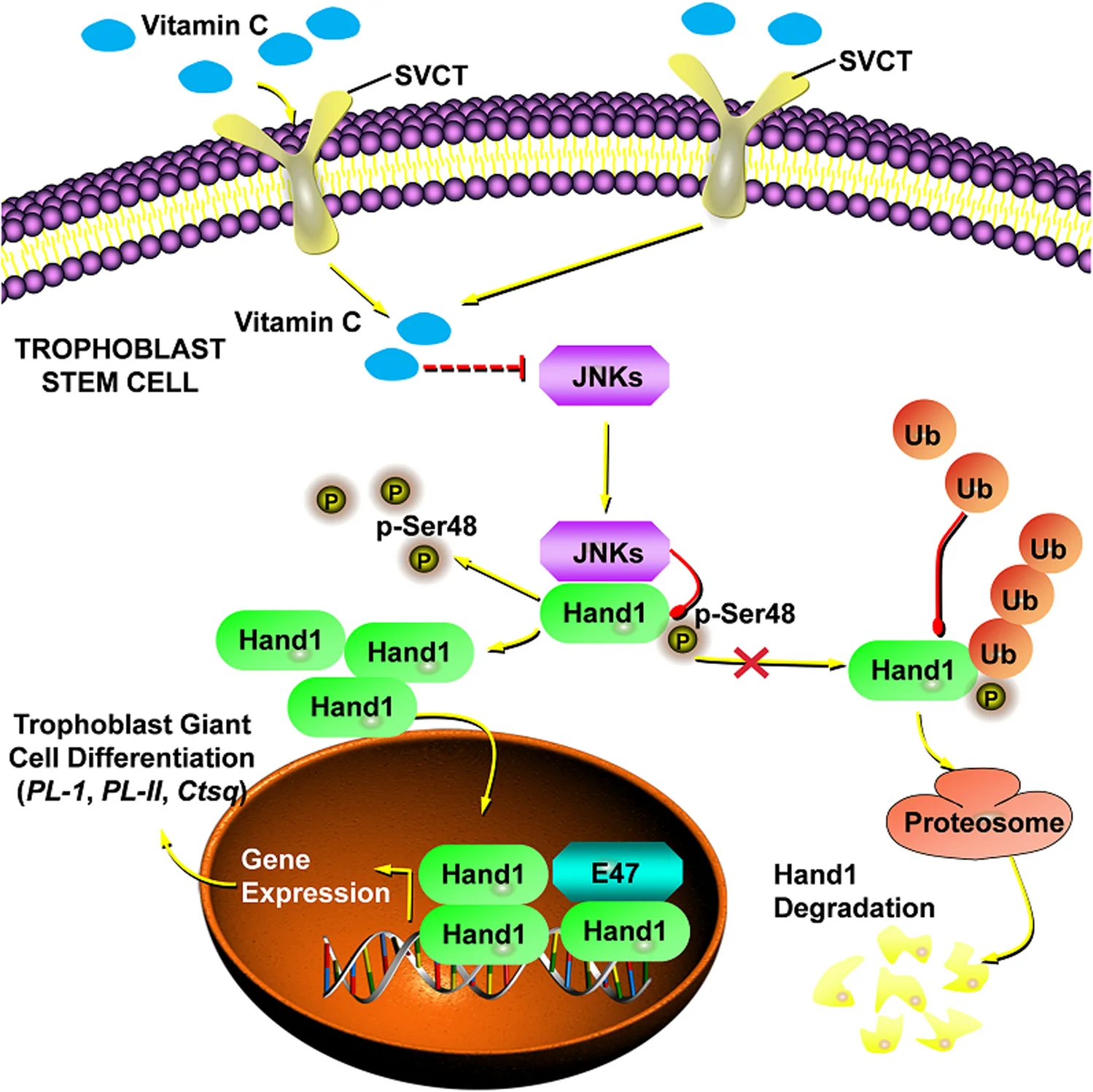

**Supplementary Information:**

The online version contains supplementary material available at 10.1007/s00018-024-05345-6.

## Introduction

Placenta is the first organ of embryonic origin and connects the fetus to the maternal body to allow the exchange of nutrients, blood and gases [1, 2]. Mouse placental development initiates at embryonic day (E) 3.5 when the formation of the trophectoderm (TE) layer envelops the blastocyst, and then the labyrinth layer begins to form at E8.5 when the allantois attaches to the chorion, followed by the chorionic branching, fetal vasculogenesis, and the intricate construction of maternal circulation [3, 4]. By E14.5, the mature placenta exhibits a complex structure comprising the trophoblast giant cell (TGC) layer, spongiotrophoblast (SpT) layer, and labyrinthine (LB) layer. Within this framework, the TGC layer comprises multiple TGC subtypes, while the SpT layer consists of SpTs and glycogen trophoblast cells [1, 3]. The LB layer serves as the core site for nutrient uptake and waste elimination, characterized by a complicated network of maternal blood sinus (MBSs) and fetal blood vessels (FBVs) segregated by a placental barrier consisting of four cellular strata: sinusoidal TGCs (S-TGCs) lining the maternal lacunae, layer I and II syncytiotrophoblasts (ST-Is and ST-IIs) forming syncytial bilayers, and endothelial cells (ET) enveloping the FBVs [1, 3].

Trophoblast stem cells (TSCs) represent the progenitors to a spectrum of differentiated trophoblast lineages within the placenta [5]. Under specific differentiation conditions, TSCs undergo robust spontaneous differentiation into distinct TGC subtypes expressing heart and neural crest derivatives expressed 1 (Hand1), serving as a pivotal transcriptional factor across all TGC subtypes [6]. These individualized TGC subtypes manifest characteristic gene expression profiles: P450 cholesterol sidechain cleavage enzyme (*P450scc*) is restrictedly expressed in parietal TGCs (P-TGCs); Placental lactogen-1 (*Pl-1*) and cathepsin Q (*Ctsq*) denote unique markers for P-TGCs and S-TGCs, respectively. Placental lactogen-2 (*Pl-2*) demonstrates expression in P-TGCs, maternal canal-associated TGCs (C-TGCs) and S-TGC, while proliferin (*Plf*) expression is confined to P-TGCs, spiral artery-associated (SpA-TGCs), and C-TGCs. Besides, TSCs possess the capability to differentiate into the SpTs expressing the markers of mammalian Achaete-scute homolog 2 (*Ascl2*) and trophoblast-specific protein α (*Tpbpα*), and into ST-Is and ST-IIs expressing the markers of syncytin-A (*SynA*) and syncytin-B (*SynB*), respectively [5]. The cultivation of TSCs into trophoblast lineages within a controlled environment renders this ex vivo system invaluable for exploring the regulatory mechanisms guiding TSCs differentiation across health and disease spectra.

Vitamin C (VC), an indispensable nutrient, plays diverse roles in cellular functions including anti-oxidation processes, metabolic reactions, and stem cell differentiation [7, 8]. Emerging evidence highlights the detrimental implications of maternal VC deficiency on fetal development [9]. The cellular uptake of VC is governed by non-overlapping sodium-dependent vitamin C transporters (SVCTs) and glucose transporters (GLUTs) specialized for transporting both the reduced (ascorbic acid) and oxidized form (dehydroascorbic acid, DHA) of VC across biological membranes [7, 8]. Notably, SVCT1 primarily localizes to epithelial surfaces within the intestine and renal for bulk transport functions, while SVCT2 facilitates the selective VC uptake in metabolic active tissues such as neurons, endocrine organs, bone, and placenta [7, 8]. Interestingly, phenotype analysis of *SVCT2*-deficient mouse offspring and placenta display starkly diminished or absent VC levels in blood and tissues, with prenatal supplementation in pregnant females failing to elevate blood VC concentrations in *SVCT2*-deficient pups, leading to perinatal lethality marked by respiratory failure and intraparenchymal brain hemorrhage [10, 11]. Subsequent assessments indicate that *SVCT2*-deficient pups encountered severe cortical hemorrhage, brain stem hemorrhage and cell loss, heightened apoptotic rates and disruption of the basement membrane in the fetal brain [12]. In addition, SCVT2-mediated VC uptake proves essential for human placental steroid and peptide hormone synthesis, suggesting the potential importance of VC in the maintenance of human pregnancy [13, 14]. All these findings underscore the vital significance of placental VC transport in maternal sustenance.

During human pregnancy, serum VC levels drop from 62.5 μM to approximately 28.4 μM after the first 4 months of gestation, further declining to around 19.9 μM before parturition [13, 14]. Moreover, the reduction of circulating VC levels in pregnant women is closely associated with the development of pre-eclampsia [15]. Therefore, the recommendation for pregnant women is to uphold a dietary VC allowance of at least 200 mg/day, ensuring serum concentrations stabilize around 60 μM, a value similar to the maximal velocity (*V*max) of the human SVCT2 transporter [8, 16, 17]. Given the relatively low serum VC concentrations experienced during pregnancy along with the substantial production of free radicals within the placenta, the supplementation of VC emerges as a promising strategy for mitigating the risk of preeclampsia and accompanying pregnancy complications [2, 18, 19]. Regrettably, the accumulating clinical evidence fails to substantiate either the singular administration of VC or its combination with other anti-oxidants during pregnancy as efficacious preventative measures against fetal or neonatal mortality, intrauterine growth retardation, preterm birth, pre-eclampsia, or miscarriage occurrences [20–23].

Despite the inconclusiveness of VC supplementation in preventing pregnancy-related disorders, the specific role of VC deficiency in placentation and maintenance of pregnancy remains completely enigmatic. In the present study, we demonstrate that VC deficiency triggers the activation of c-Jun N-terminal kinase (JNK) signaling, resulting in the direct phosphorylation and subsequent destabilization of Hand1, a bHLH transcription factor crucial for TGC lineage development, consequently impeding successful placentation and the continued maintenance of pregnancy.

## Results

### VC orchestrates TSCs differentiation into multiple TGC subtypes and upregulates Hand1 protein expression

To explore the role of VC on TSCs differentiation, the primary mouse TSCs underwent culture in a differentiation medium with varying VC concentrations (10, 30, or 100 μM) for 3 days. Cell viability assessments using the MTT assay unveiled a dose-dependently enhancement in cell viability with VC treatment (Fig. S1A). Morphological assessments through light microscopy revealed that TSCs cultured in differentiation medium displayed larger cell sizes and blurred boundaries compared to those cultured in proliferation medium, indicating a transition toward TGC differentiation. Notably, the presence of VC further amplified these morphological alterations, yielding cells with enhanced irregularities and a network-like appearance, indicative of an advanced stage of TGC differentiation (Fig. S1B).

**Fig. 1:**
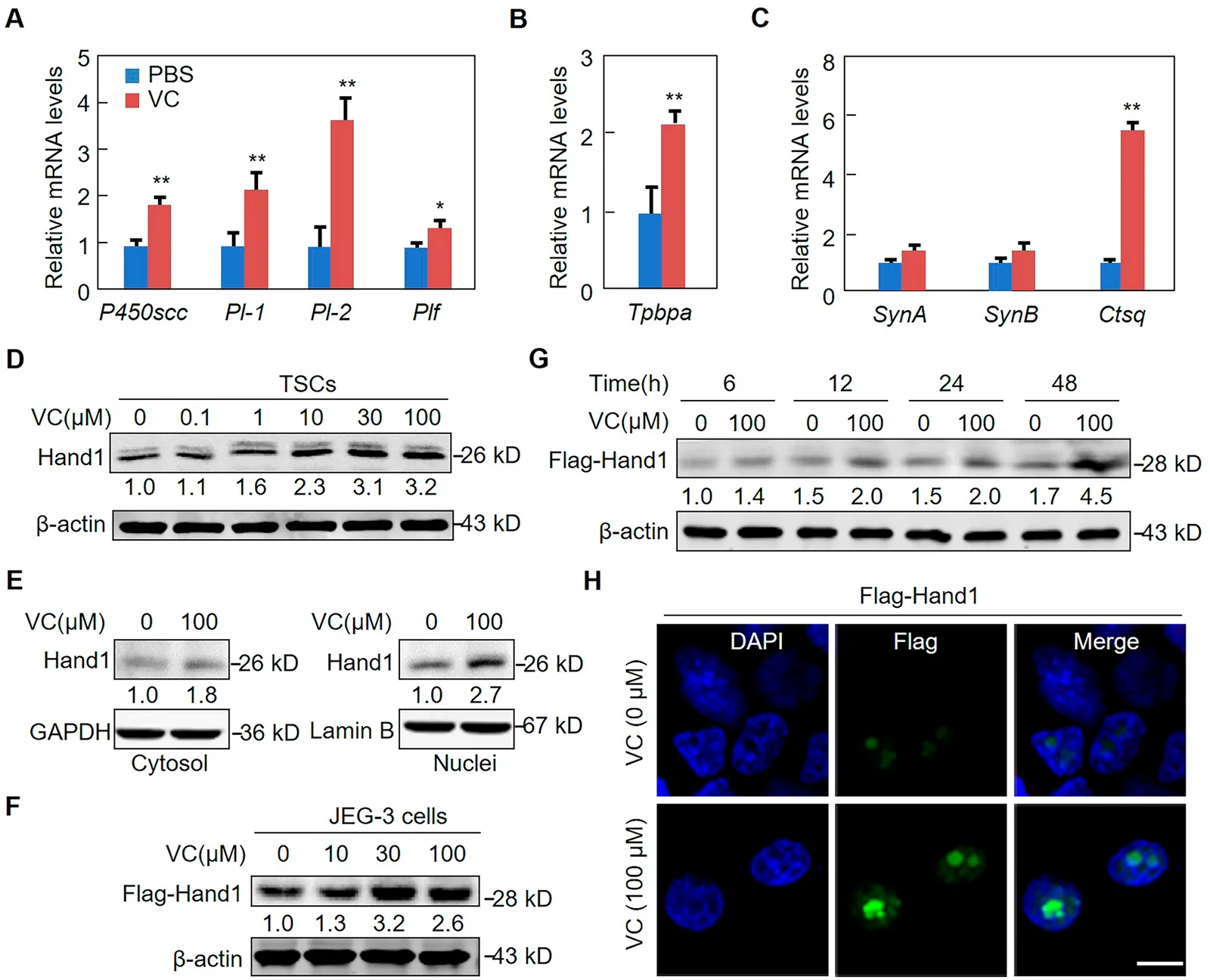
VC induces the TSC differentiation and Hand1 expression. **A-C** Quantitative RT-PCR assays for trophoblastic differentiation markers in the primary TSCs incubated in the differentiation medium with or without VC at 100 μM for 3 days. P-TGC markers, *P450scc*, *Plf*, *PL-1*, *PL-2*; S-TGC marker *Ctsq*; SpTs marker *Tpbpa*; ST-Is and ST-IIs markers *SynA* and *SynB*, respectively. **D**-**E** Western analyses of Hand1 in whole primary TSCs or in cytosolic versus nuclear fractions of primary TSCs incubated in the differentiation medium with or without VC for 48 h. **F–H** Western and immunofluorescence analyses of the Flag tag in JEG-3 cells transfected with Flag-Hand1 and treated with VC for the indicated times or 48 h. Numerical data: mean ± SD, n = 3, *p < 0.05, **p < 0.01

Then, quantitative RT-PCR (qPCR) analysis of trophoblast-specific markers, including P-TGC markers (*P450scc*, *Plf*, *PL-1*, *PL-2*), S-TGC marker *Ctsq*, and SpTs marker *Tpbpa*, as well as ST-I marker (*SynA*) and ST-II marker (*SynB*), indicated that VC treatment robustly induced the mRNA levels of *Ctsq, PL-2, PL-1, P450scc,* and *Plf* by approximately 4.7-, 2.7-, 1.2-, 0.8- and 0.4-fold, respectively, compared to vehicle treatment (Fig. 1A–C). Additionally, VC treatment enhanced *Tpbpα* mRNA levels by 1.3-fold, with no discernible impact on *SynA* or *SynB* mRNA levels (Fig. 1B, C). All these findings collectively highlight VC as a potent driver of TSCs differentiation, particularly into diverse TGC subtypes.

Given the pronounced enhancement of TGC markers in response to VC treatments, and Hand1's integral role in the differentiation of all TGC subtypes [5], the Hand1 mRNA and protein levels in TSCs following VC exposure were evaluated using qPCR and Western blot analysis. We found that VC treatments did not alter the *Hand1* mRNA levels but increased the Hand1 protein expression in a dose-dependent manner, particularly treated with 30 and 100 μM VC (Fig. 1D and Fig. S1C). Subsequent Western blot analysis of cytosolic and nuclear fractions of Hand1 protein corroborated heightened cytosolic and nuclear Hand1 levels post-VC treatments (Fig. 1E). Likewise, further assessments conducted in human trophoblast-like choriocarcinoma JEG-3 cells or HEK293T cells (293T) transiently transfected with Flag-tagged Hand1 (Flag-Hand1) consistently elicited a dose- and time-dependent increase in exogenous Hand1 protein levels upon VC treatments (Fig. 1F, G) and Fig. S1D). And immunofluorescence analysis of Flag-Hand1 in JEG-3 cells further demonstrated increased exogenous Hand1 levels in both the cytoplasm and nucleus in the presence of VC (Fig. 1H). The above results underscore the pivotal role of VC in enhancing Hand1 protein expression during TSCs differentiation.

Basic HLH factors typically form homodimers or heterodimers to induce TGC differentiation, and induction of TSCs differentiation into TGCs requires the formation of Hand1 homodimers for transcription regulation [24]. To determine if VC modulates the formation of Hand1 homodimers, a co-immunoprecipitation assay was performed in JEG-3 cells co-transfected with HA-Hand1 and Flag-Hand1 and treated with or without VC. Western assays using a Flag antibody indicated that VC treatment did not affect the Hand1 homodimerization but rather augmented the quantity of Hand1 homodimers by eliciting an increase in Hand1 protein expression (Fig. S1E). These findings suggest VC induces TSCs differentiation into TGC subtypes primarily by upregulating Hand1 expression rather than modulation of its dimerization capacity.

### VC suppresses JNK signaling to enhance Hand1 expression for TSCs differentiation into TGCs

Since VC is recognized for its potent antioxidant properties in cellular functions, we investigated whether VC induces TSCs differentiation into TGCs by leveraging its antioxidative capabilities. TSCs were respectively incubated with various antioxidants such as N-acetyl cysteine (NAC), *α*-tocopherol (VE), glutathione (GSH), and lipoic acid (LA), and the impacts of these antioxidants on *Ctsq* mRNA expression were assessed using qPCR analyses, juxtaposing their induction effects with that of VC. Intriguingly, while VC treatment notably elevated *Ctsq* mRNA levels, other antioxidants like NAC, VE, GSH, and LA exhibited no noteworthy impact on basal *Ctsq* mRNA expression. Conversely, simultaneous treatment of VC with different oxidants such as H_2_O_2_ or Diethyl maleate (DEM) failed to counteract VC’s enhancement of *Ctsq* mRNA expression (Fig. S2). Based on these results, we speculated that VC induces TSCs differentiation into TGCs independent of its anti-oxidative property.

**Fig. 2:**
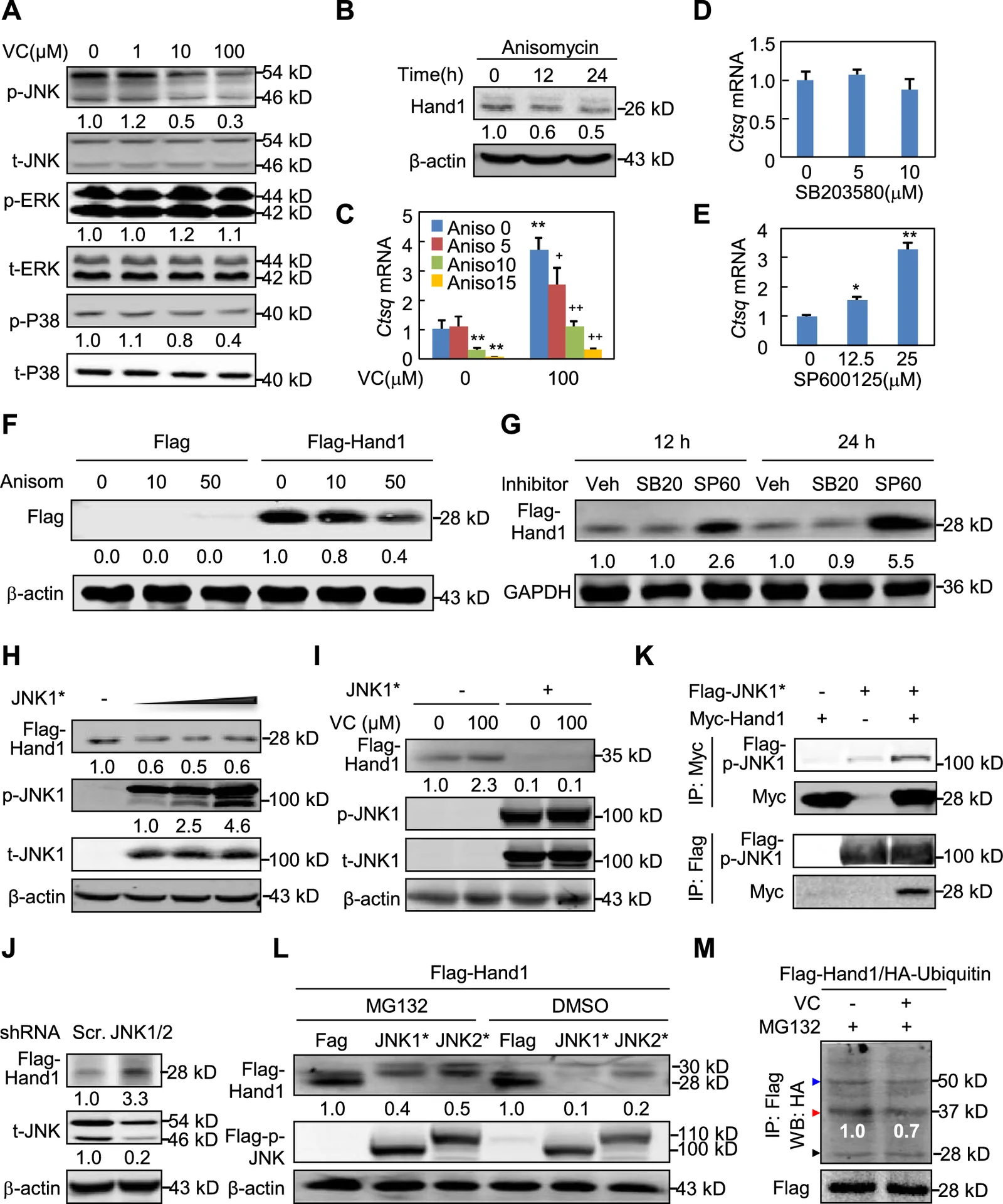
VC inactivates JNK to stabilize Hand1. **A** Western blot analysis of MAPK activity in the primary TSCs incubated with or without VC for 30 min. **B** Western analyses of Hand1 in the primary TSCs incubated with or without anisomycin at 10 μM. **C-E** Quantitative RT-PCR assays of *Ctsq* in the primary TSCs incubated with or without anisomycin, SB203580 or SP600125 and in the presence or absence of VC for 72 h. **F**-**G** Western analyses of Flag tag for JEG-3 cells transfected with Flag-Hand1 and treated with anisomycin for 24 h or 10 μM SB203580(SB20)/25 μM SP600125(SP60) for 12 or 24 h. **H-I** Western analyses of the Flag tag in JEG-3 cells transfected with Flag-Hand1 and JNK1* and treated with or without VC for 24 h. **J** Western analyses of the Flag tag in JEG-3 cells transfected with Flag-Hand1 for 48 h, following the infection with Scramble- or JNK1/2-shRNA-expressing lentiviruses. **K** Western analyses of Flag-p-JNK1 for the immunocomplex precipitated by a Myc or Flag antibody in JEG-3 cells transfected with Flag-JNK1* and Myc-Hand1. **L** Western analyses of Flag tag (15% SDS-PAGE) in JEG-3 cells transfected with Flag-JNK1*/2* and Flag-Hand1 and treated with MG132 at 10 μM or DMSO for 24 h. **M** Western analyses of HA tag for the immunocomplex precipitated by a Flag antibody in JEG-3 cells transfected with Flag-Hand1 and HA-ubiquitin and treated with or without 100 μM VC in the presence of 10 μM MG132. Numerical data: mean ± SD, *n* = 3, *^,+^*p* < 0.05, **^,++^*p* < 0.01

In a previous study, it was demonstrated that fibroblast growth factor 4 (FGF4)-mediated maintenance of TSCs stemness relies on MAPK kinase kinase (MEKK4)-triggered JNK signaling [25], whereas our previous research revealed that DHA, an oxidized form of VC, could inhibit JNK phosphorylation to regulate steroidogenesis in human trophoblast-like JAR cells [26], hinting at potential effects of VC on JNK signaling. To identify the impact of VC on TSCs’ MAPK signaling, Western analyses revealed that exposure of TSCs to VC for 30 min led to a significant reduction in phosphorylated JNK (p-JNK) and P38 (p-P38) levels by up to 30% and 40%, respectively, while leaving phosphorylated extracellular signal–regulated kinases 1/2 (p-ERK) unaffected (Fig. 2A). In contrast, anisomycin, a dual JNK and P38 agonist, time-dependently negated Hand1 protein levels and dose-dependently diminished VC-induced *Ctsq* mRNA in TSCs (Fig. 2B, C). Noteworthy, the JNK inhibitor SP600125 dose-dependently increased *Ctsq* mRNA levels in TSCs, whereas the P38 inhibitor SB203580 had no discernible effect on *Ctsq* mRNA levels (Fig. 2D, E). Furthermore, anisomycin and SP600125 markedly lowered and raised Flag-Hand1 expression in JEG-3 cells, respectively, whereas SB203580 had no impact on Flag-Hand1 levels (Fig. 2F, G). These results suggest that JNK signaling, rather than P38 and ERK1/2 signaling, plays a pivotal role in VC-induced Hand1 protein expression and the differentiation of TSCs into TGCs.

To further elucidate the involvement of JNK signaling in VC-induced Hand1 expression, gain- and loss-of-function experiments of JNK signaling were executed by transfecting JEG-3 cells with a constitutively active form of JNK1 (JNK1*) plasmid created through a fusion protein of Jun N-terminal kinase kinase 2 (JNKK2) and JNK1, as well as lentiviral transduction to knock down JNK1/2. We found that JNK1* overexpression significantly diminished both basal and VC-induced Flag-Hand1 protein expression levels in JEG-3 cells, while JNK1/2-shRNA-expressing lentiviruses, which reduced the JNK1/2 by approximately 80%, increased Flag-Hand1 protein levels by 2.3-fold, compared with Scramble-shRNA-expressing lentiviruses (Fig. 2H–J). Then, to probe the potential interaction between Hand1 and JNK1*, co-immunoprecipitation experiments were carried out using cell lysates from JEG-3 cells transiently transfected JNK1* alone, Myc-Tag Hand1 (Myc-Hand1) alone, or both plasmids together. We found that cells expressing either Myc-Hand1 or JNK1* in isolation showed limited JNK1*, whereas co-expression of both proteins exhibited abundant JNK1* (Fig. 2K). Therefore, JNK1* physically interacts with and negates the Hand1 protein.

To ascertain whether JNKs negate the Hand1 protein through proteasomal degradation pathway, JEG-3 cells expressing Flag-Hand1 and JNK1*/JNK2* were treated with MG132, a proteasome inhibitor, and Western analyses on a 15% SDS-PAGE were utilized to assess potential shifts in Hand1 band post-JNK-mediated phosphorylation. The results revealed that both JNK1* and JNK2* robustly reduced Flag-Hand1 levels in JEG-3 cells, while JNK1* exhibited stronger effects than JNK2* (Fig. 2L). Moreover, JNK1* and JNK2* consistently caused an apparent upward shift in Flag-Hand1 bands, while MG132 treatments did not alter the band shift but effectively reversed the Hand1 reduction induced by JNK1* or JNK2* (Fig. 2L), suggesting a direct involvement of JNK1/2 in the degradation of Hand1 through the proteasomal manner. To confirm this notion, we used MG132 and VC to block the proteasomal degradation and JNK activity, respectively, in JEG-3 cells transfected with Flag-Hand1 and HA-tagged ubiquitin (HA-Ubiquitin). Co-immunoprecipitation utilizing a Flag antibody followed by Western blotting with a HA antibody was performed and the results indicated that VC-treated cells exhibited a 50% reduction in ubiquitinated Hand1 with a molecular weight prediction of 37 kDa (band #1) and a 30% decrease in ubiquitinated Hand1 with a molecular weight prediction of 50 kDa (band #2) compared to control cells (Fig. 2M). These findings suggest that VC inactivates JNK to block the ubiquitination-mediated proteasomal degradation of Hand1.

### JNK phosphorylates Ser48 of Hand1 to facilitate its degradation

**Fig. 3:**
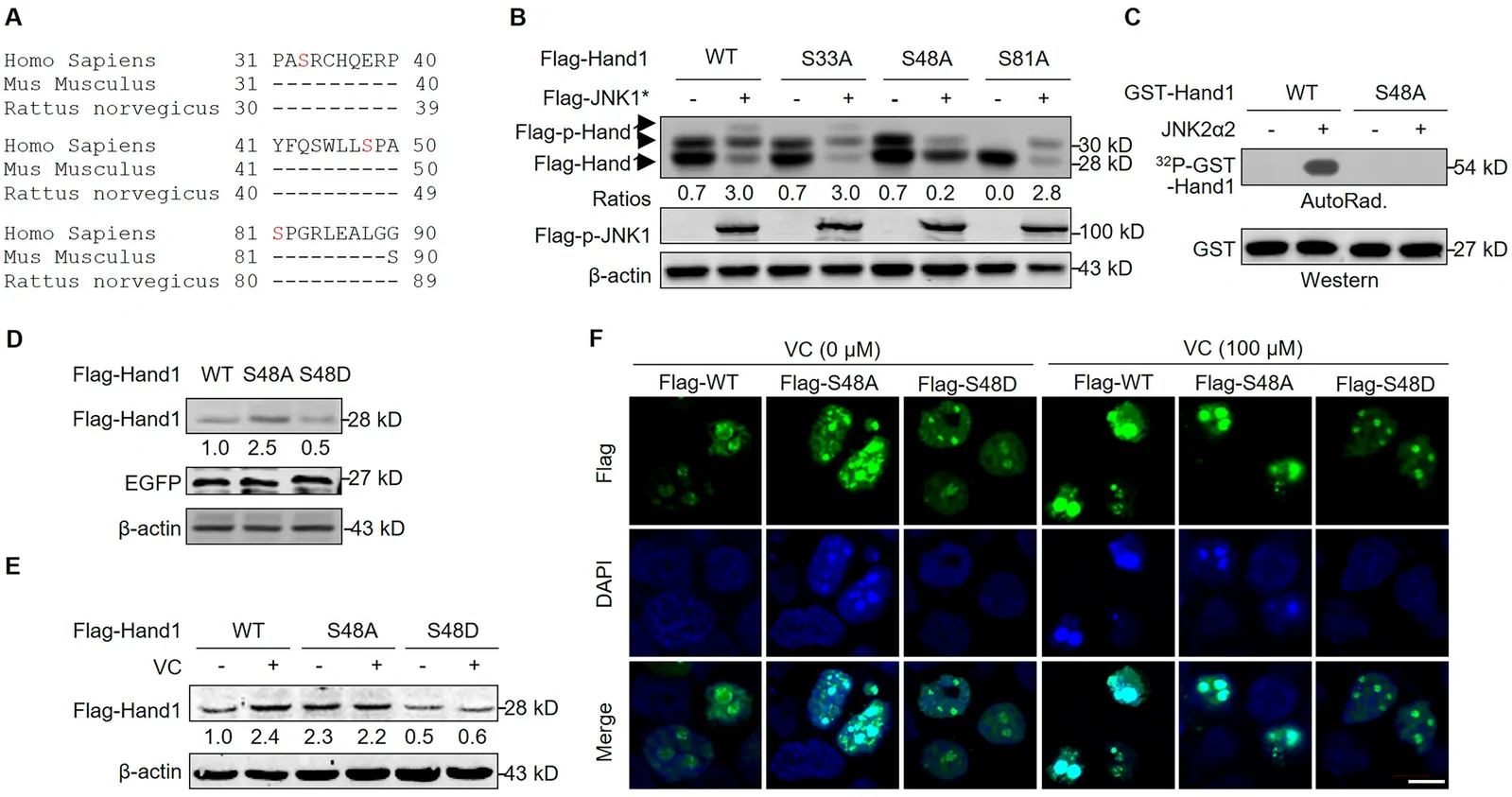
JNK directly phosphorylates Hand1 at Ser48 to destabilize Hand1. **A** The conserved sites of Hand1 for potential phosphorylation by MAPK. **B** Western analyses of Flag tag (15% SDS-PAGE) in JEG-3 cells transfected with Flag-Hand1 variants and Flag-JNK1*. Ratios: phosphorylated versus non-phosphorylated Flag-Hand1. **C** In vitro phosphorylation of GST-Hand1 variants by JNK2α2. Autoradiography signals were normalized to levels of GST-Hand1 variants. **D-E** Western analyses of Flag tag in JEG-3 cells transfected with Flag-Hand1 variants with or without the control EGFP-expressing vector in the presence or absence of 100 μM VC for 48 h. **F** Immunofluorescence assays of Flag tag in JEG-3 cells transfected with Flag-Hand1 variants and treated with or without VC for 48 h

The interaction between JNK and Hand1 prompted a direct exploration of JNK's Hand1 phosphorylation. Utilizing the Group-based Prediction System (GPS), potential JNK phosphorylation sites on Hand1 were identified. Analysis of the Hand1 protein sequence unveiled high or low prediction scores for Ser33, Ser48, and Ser81 sites, as well as for Ser98, Thr107, and Ser109 sites, all conserved among human, mouse, and rat (Fig. 3A). To further investigate significance of these sites, Flag-Hand1 variants were generated with mutations at the consensus serine or threonine residue (Ser and Thr to Ala) individually (S33A, S48A, S81A, S98A, T107A, and S109A) and evaluated for their response to JNK1* in JEG-3 cells through Western analyses on a 10% SDS-PAGE. In the absence of JNK1*, Flag-Hand1 levels for the wild-type (WT) resembled those of S33A, S81A, S98A, T107A or S109A variants, but were 50% lower than the S48A variant; in the presence of JNK1*, Flag-Hand1 levels in WT persisted similar to S33A, S81A, S98A, T107A, or S109A variants, but were 85% lower compared to the S48A variant (Fig. S3A and B). Thus, Ser48 emerged as a key amino acid residue in mediating JNK-induced destabilization of Hand1.

Subsequently, Western assays were conducted on a 15% SDS-PAGE to assess phosphorylated and non-phosphorylated Flag-Hand1 bands following JNK1* overexpression. We found that the S33A and S48A variants displayed comparable levels of phosphorylated and non-phosphorylated Flag-Hand1 levels similar to the WT in the absence of JNK1*. Conversely, the S81A variant showed an unexpected elimination of phosphorylated Flag-Hand1 band while maintaining consistent levels of non-phosphorylated Flag-Hand1 band (Fig. 3B). In the presence of JNK1*, the WT, S33A, and S81A variants exhibited nearly identical ratios between phosphorylated and non-phosphorylated Flag-Hand1 levels (3:1), whereas the S48A variant markedly shifted this ratio to 1:5. Notably, both the S48A and S81A variants seemingly eliminated the highest phosphorylated band of Flag-Hand1 (Fig. 3B). These observations imply that while Ser81 on Hand1 protein may potentially serve as a phosphorylation site for kinases other than JNK, Ser48 remains crucial as a JNK-specific phosphorylation site.

To ascertain whether JNK could directly phosphorylate Hand1 at Ser48, an in vitro phosphorylation assay was conducted employing a commercially available active JNK2α2 with purified recombinant glutathione S-transferase (GST)-tagged WT Hand1 or its S48A mutant (Fig. S3C). Following SDS-PAGE and autoradiography, the WT Hand1 exhibited notable ^32^P incorporation, while the S48A variant showed no ^32^P uptake even in the presence of active JNK2α2 (Fig. 3C, top). Subsequent Western blotting with the GST antibody confirmed the ^32^P-labeled band corresponded to the GST-Hand1 variants, displaying consistent levels across reactive mixtures (Fig. 3C, bottom), indicative of direct phosphorylation of Hand1 by JNK at Ser48.

To evaluate the significance of Ser48 phosphorylation of the Hand1 protein, we introduced mutations by substituting the serine residue at position 48 of Hand1 with either the phosphomimetic aspartate (D) to stimulate activation of Hand1 or with alanine to mimic inactivation of Hand1, which was transfected into JEG-3 cells alongside an enhanced green fluorescent protein (EGFP) vector to monitor transfection efficiency. Western analyses confirmed consistent EGFP expression levels, ensuring uniform transfection effectiveness across samples. Notably, cells transfected with S48A and S48D mutants respectively exhibited a 150% increase and a 50% decrease in Flag-Hand1 levels compared to those transfected with the WT Hand1 (Fig. 3D). Moreover, treatment with 100 μM VC elicited a 1.4-fold elevation in Flag-Hand1 expression in cells transfected with WT construct, while no significant alteration was observed in cells transfected with the S48A or S48D variants (Fig. 3E). In parallel, immunofluorescent analysis of JEG-3 transfected with these Flag-Hand1 variants using Flag antibody revealed that, under basal conditions, cells transfected with WT Hand1 exhibited a relatively modest fraction of Hand1 in cytoplasm and an exclusive nucleus level; the Hand1 S48A mutation resulted in a much higher nucleus fraction compared to WT, whereas the Hand1 S48D mutation led to notably reduced levels of Hand1 in both the nucleus and cytoplasm. Moreover, VC stimulation promoted increased levels of WT Hand1 in both compartments, but not in those transfected with S48A or S48D variants (Fig. 3F). These in vitro findings underscore the crucial role of Ser48 in Hand1 for the VC-mediated enhancement of Hand1 protein expression.

### The VC/JNK/Hand1 pathway induces TGC differentiation in murine placentas

Since VC could suppress JNK activity to stabilize Hand1 expression, and the differentiation of TSCs into all TGC subtypes relies on the contribution of Hand1 protein [24, 27, 28], we further elucidated the mechanisms of VC/JNK/Hand1 pathway on TGC differentiation in E8.5 placentas by leveraging multiple strategies, including VC deficiency, lentivirus-mediated knockdown of JNK or overexpression of Hand1 mutants. We first generated global *L-gulono-γ-lactone oxidase* (*Gulo*^*−/−*^) knockout mice, genetically incapable of synthesizing VC, and raised on tap water containing 4 g/L of VC for normal growth [29]. We then crossed the *Gulo*^*−/−*^ females with the *Gulo*^*−/−*^ males to generate the *Gulo*^*-/-*^ placentas, and pregnant *Gulo*^*−/−*^ females were deprived of VC post-plug identification (E0.5). HPLC analysis of maternal serum VC concentrations indicated VC deprivation resulted in a progressive decline in serum VC concentrations, dropping from 53.5 μM from E0.5 to approximately 11.5 μM by E8.5 (Fig. S4A).

**Fig. 4:**
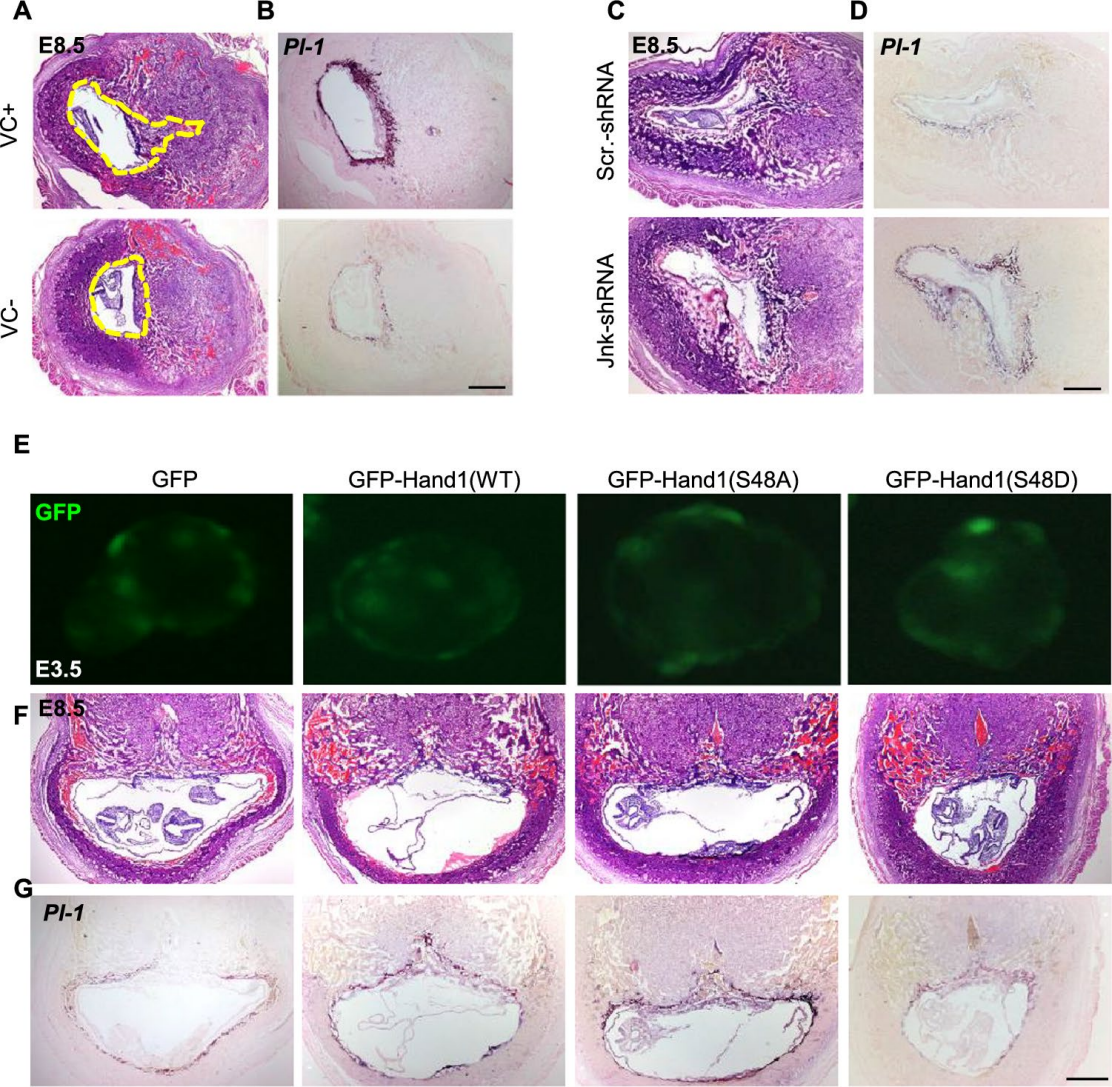
*Pl-1* expression in the implantation sites of E8.5 placentas. **A-B** HE staining (**A**) and in situ hybridization assays of *Pl-1* (**B**) in the implantation sites of E8.5 *Gulo*^*−/−*^ placentas supplemented with or without VC. **C-D** HE (**C**) and in situ hybridization assays of *Pl-1* (**D**) in the implantation sites of E8.5 placentas infected with Scramble- or JNK1/2-shRNA expressing lentiviruses. **E** GFP expression in the E3.5 blastocysts 8 h after infection with GFP-bearing and Hand1 variants-expressing lentiviruses. **F-G** HE staining (**F**) and in situ hybridization of *Pl-1* (**G**) in the implantation sites of E8.5 placentas infected with GFP-bearing and Hand1 variants-expressing lentiviruses

To investigate the VC deficiency on early development of placentation, pregnant *Gulo*^*−/−*^ females were dissected at E8.5, and conceptuses were then subjected to cryosection. And HE staining and in situ hybridization with *Pl-1* probe, a specific marker for P-TGC, which also serves as an indicator of Hand1 activity [6], were carried out. The results showed that in the presence of VC supplementation, *Pl-1*-positive P-TGCs distinctly lined implantation sites, demarcating the maternal decidua from the ectoplacental cone. Conversely, in the absence of VC supplementation, *Pl-1*-positive P-TGCs were faintly arrayed along implantation sites, fuzzily separating the maternal decidua from the ectoplacental cone (Fig. 4A, B), indicating VC insufficiency impairs P-TGCs differentiation at the implantation sites, potentially by inhibiting Hand1 activity.

To confirm the importance of JNK activity in TGC differentiation in vivo, E3.5 blastocysts were transduced with lentiviruses carrying green fluorescent protein (GFP) along with Jnk1/2-shRNA to specific knockdown JNK1/2 protein in trophectoderm, followed by blastocyst transplantation. Immunofluorescent analysis of GFP expression at E3.5 blastocysts indicated the lentiviruses were efficiently delivered to the trophectoderm, excluding the inner cell mass (ICM) (Fig. S4B). Moreover, Western blot analysis of the whole E16.5 placentas showed that Jnk1/2-shRNA, which reduced the JNK2 and JNK1 expression by approximately 36% and 63%, respectively, resulted in approximately a 2.5-fold elevation in the Hand1 protein levels compared to Scramble-shRNA (Fig. S4C). Consistently, in situ hybridization of *Pl-1* indicated that Jnk1/2-shRNA robustly amplified the number of *Pl-1*-positive P-TGCs at E8.5 placentas, distinctly lining the implantation sites and clearly separating the maternal decidua from the ectoplacental cone, in contrast to Scramble-shRNA (Fig. 4C, D). These results collectively demonstrate that JNK inactivation enhances TGC differentiation in murine placentas by increasing both Hand1 protein levels and activity.

Then, the in vivo significance of Hand1 mutants on TGC differentiation was assessed by transducting E3.5 blastocysts with GFP-carrying lentiviruses expressing Hand1(WT)-, Hand1(S48A)- or Hand1(S48D), respectively, followed by transplantation. Immunofluorescent analysis of GFP expression of E3.5 blastocysts prior to transplantation revealed that the GFP-carrying Hand1 variants were effectively integrated and uniformly expressed in the trophectoderm (Fig. 4E). Moreover, HE staining and in situ* Pl-1* hybridization revealed that overexpression of Hand1(WT) moderately enhanced the number of *Pl-1*-positive P-TGCs; overexpression of Hand1(S48A) robustly enhanced the number of *Pl-1*-positive P-TGCs at E8.5 implantation sites, whereas overexpression of Hand1(S48D) notably reduced the number of *Pl-1*-positive P-TGCs compared to Hand1(WT) overexpression (Fig. 4F, G), suggesting S48A and S48D mutations in Hand1 have positive and negative effects on TGC differentiation. Overall, these in vivo findings highlight that VC inactivates JNK signaling to increase the Hand1 protein level and activity, thereby facilitating TGC differentiation in placentas.

### VC deficiency causes the failure of maintenance of pregnancy

**Fig. 5:**
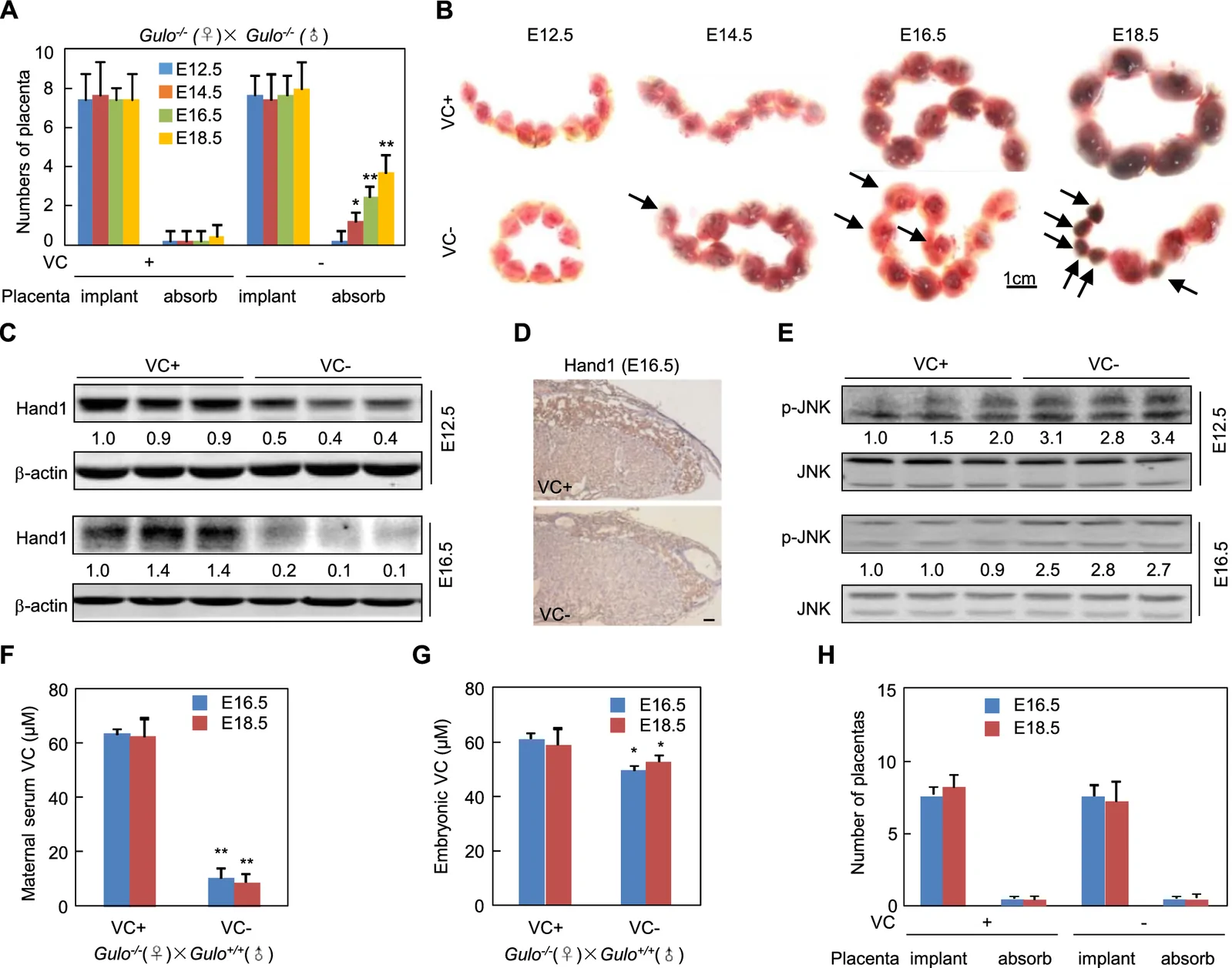
VC deficiency fails to maintain the pregnancy. **A-B** Numbers of the implanted and absorbed placentas and embryos from the pregnant *Gulo*^*−/−*^ females (crossed with *Gulo*^*−/−*^ males) supplemented with or deprived of VC at 4 g/L in the tap water from E0.5. Specifically, we analyzed a total of 245 placentas, distributed as follows: 61 placentas each at E12.5, E14.5, and E16.5, and 62 placentas at E18.5. In the VC-deficient group, the incidence of affected placentas was approximately 3% (*n* = 1) at E12.5, 7% (*n* = 2) at E14.5, 19% (*n* = 6) at E16.5, and 38% (*n* = 12) at E18.5. In contrast, the VC-supplemented group showed significantly lower incidences: 3% (*n* = 1) at each of E12.5, E14.5, and E16.5, and 7% (*n* = 2) at E18.5. Arrows: absorbed placentas and embryos. **C-E** Western and immunohistochemistry assays of Hand1 or p-JNK in the viable placentas with the same treatments as described in (**A** and **B**). **F-G** VC concentrations in the sera and embryos of pregnant *Gulo*^*−/−*^ females (crossed with WT males) supplemented with or deprived of VC at 4 g/L in the tap water from E0.5. **H** Numbers of the implanted and absorbed placentas from the pregnant *Gulo*^*−/−*^ females as described in (**F** and **G**). Numerical data: mean ± SD, *n* = 6 ~ 8, *p < 0.05, **p < 0.01

Subsequently, we dissected the significance of VC in sustaining pregnancy by studying the midgestational (E12.5 and E14.5) to late-gestational stages (E16.5 and E18.5) of pregnant *Gulo*^*-/-*^ females carrying *Gulo*^*-/-*^ progeny. We found VC withdrawal since plug identification (E0.5) led to a substantial reduction of maternal serum VC levels by approximately 94% persisting through E12.5~E18.5, in comparison to 4 g/L VC supplementation (Fig. S4D). Then, the effects of VC deficiency on fetal and placental development were first assessed by determining the placental and fetal numbers and the corresponding results unveiled that VC deprivation in the pregnant *Gulo*^*-/-*^ females did not significantly alter the numbers of implanted embryos and placentas, however, VC deficiency markedly escalated the incidence of atrophic and absorbed placentas and embryos by approximately 7%, 19%, and 38% at E14.5, E16.5, and E18.5, respectively, exhibiting no significant effect at E12.5, as compared to VC supplementation (Fig. 5A, B). Furthermore, VC deficiency consistently reduced the weights of both viable placentas (data not shown) and embryos by around 10%, 22%, and 30% at E14.5, E16.5, and E18.5, respectively (Fig. S5B-D). Consequently, the inadequacy of VC fails to sustain pregnancy in *Gulo*^*-/-*^ matrices bearing *Gulo*^*-/-*^ offspring.

Osteogenic disorder (ODS) rats (genotype *od/od*) manifest a deficiency in *L-gulonolactone oxidase*, leading to a hereditary defect in VC biosynthesis, necessitating a minimum of 1 g/L of VC supplementation for normal growth [30]. To reaffirm the role of VC in supporting pregnancy, we interbred the ODS rats and withheld VC upon plug identification. Consistent with findings from *Gulo*^*−/−*^ mice, analysis of placentas and pups in ODS rats demonstrated that maternal VC deprivation notably increased the instances of atrophic and absorbed placentas and embryos by approximately 63% at E14.5 compared to VC supplementation, with no significant alterations in the quantities of implanted embryos and placentas being noted (Fig. S5A). As VC plays critical roles in the synthesis of the ovary and placental hormones that are essential for maintenance of pregnancy [13, 14], assessment of serum progesterone (P4) and estradiol (E2) levels in pregnant ODS rats at E14.5 revealed no significant difference between groups with VC supplementation or deprivation (Fig. S5B and C). Therefore, the inability of ODS rats with VC deficiency to sustain pregnancy is not attributed to insufficiency levels of P4 and E2.

Subsequent analysis concentrated on assessing the effects of VC deficiency on Hand1 and p-JNK1/2 levels in middle- and late-gestational stage of *Gulo*^-/-^ placentas using Western blot analyses, and the outcomes indicated that VC deficiency reduced the Hand1 levels in placentas by ~50% at E12.5 and ~90% at E16.5 (Fig. 5C). In parallel, immunohistochemistry staining of Hand1 expression at E16.5 placental sections depicted that VC deficiency resulted in a notable decrease in Hand1 expression globally (Fig. 5D). In contrast, VC deficiency led to a notable increase in p-JNK1/2 levels by ~104% at E12.5 and ~175% at E16.5 *Gulo*^-/-^ placentas (Fig. 5E). Thus, consistent with the aforementioned in vitro and in vivo findings, VC deficiency not only elevates p-JNK1/2 levels but also diminishes Hand1 levels in murine placentas.

To confirm the importance of fetal VC in maintaining pregnancy over maternal VC, *Gulo*^*-/-*^ females were mated with WT males to generate *Gulo*^+*/-*^ pups, with VC withheld upon plug identification. Interestingly, VC deprivation decreased the maternal serum VC levels to approximately 9 μM (versus ~60 μM) (Fig. 5F), and reduced VC levels of *Gulo*^+*/-*^ offsprings to approximately 52 μM (versus ~59 μM) at both E16.5 and E18.5 compared to VC supplementation (Fig. 5G), indicating that in contrast to *Gulo*^*-/-*^ pups, *Gulo*^+*/-*^ pups resulting from the cross of female *Gulo*^*-/-*^ and male *Gulo*^+*/*+^ mice can synthesize VC on their own despite a lack of VC supplementation to maternal *Gulo*^*-/-*^ mice. In line with the VC levels observed in the *Gulo*^+*/-*^ offspring, VC deprivation in *Gulo*^*-/-*^ matrices bearing *Gulo*^+*/-*^ pups did not result in a notable increase in the counts of the atrophic and adsorbed placentas and embryos, nor did it lead to a significant decline in the placental Hand1 expression, when compared with VC supplementation (Fig. 5Hand Fig. S5D). All these data collectively suggest that fetal VC deficiency, rather than maternal VC deficiency, is specifically linked to the failure of placentation and the maintenance of pregnancy.

### VC deficiency disrupts trophoblastic differentiation in murine placentas

**Fig. 6:**
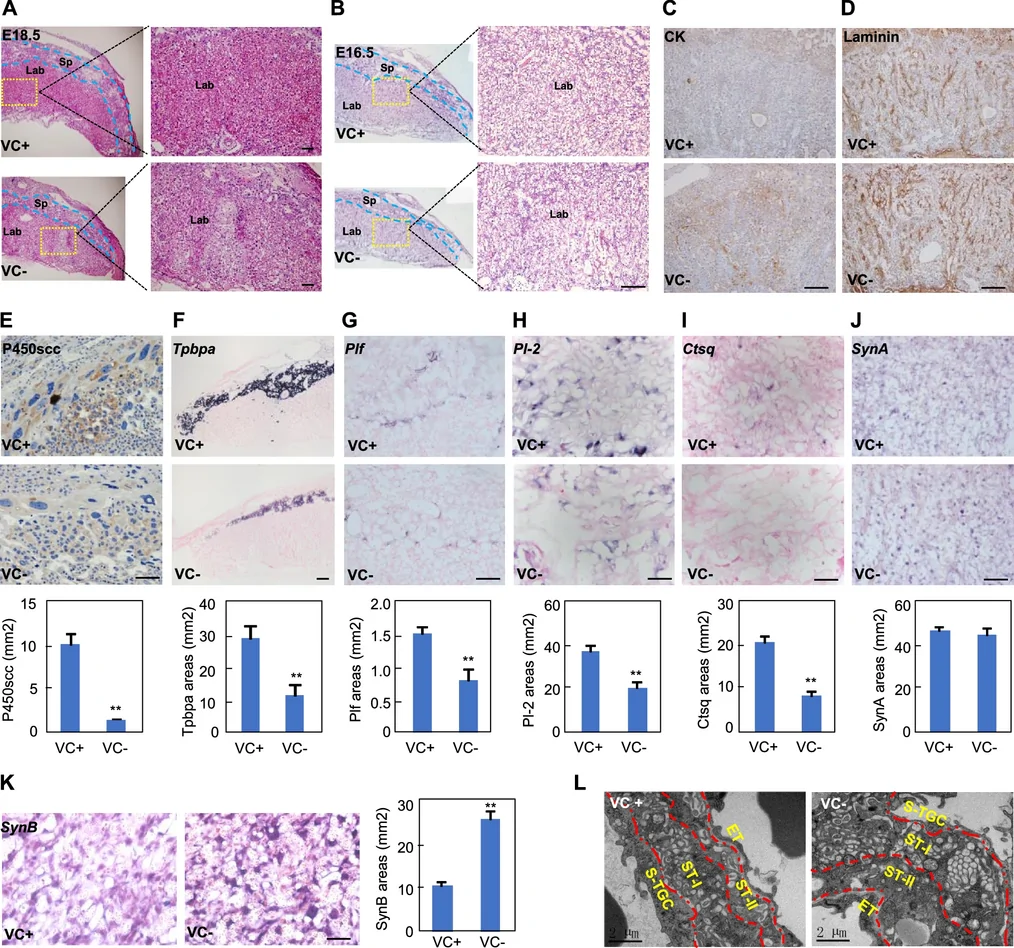
VC deficiency disrupts the differentiation of TGCs during placentation. **A-B** HE staining of E18.5 and E16.5 placental sections from the pregnant *Gulo*^*−/−*^ females (crossed with *Gulo*^*−/−*^ males) and supplemented with ( +) or deprived of (-) VC at 4 g/L in the tap water from E0.5. **C-D** Immunohistochemistry of CK and Laminin in the placental labyrinths at E16.5. **E** Immunohistochemistry of P450scc and semi-quantification in the E14.5 placental sections. **F-K** In situ hybridization assays and semi-quantification in the E16.5 placental sections. **L** Transmission electron microscopic examination of trilaminar interhemal barrier of E16.5 placentas. Numerical data: mean ± SD, *n* = 4, **p < 0.01

For a more in-depth understanding of the specific roles of VC in placentation, HE staining, immunohistochemistry staining, in situ hybridization, and transmission electron microscopic examination were performed using the viable *Gulo*^*−/−*^ placentas at E16.5 and/or E18.5. HE staining results indicated consistent reductions in the dimensions of the placental mass, junctional zone, and labyrinthine layer at both E16.5 and E18.5, with minimal changes in the decidua basalis (Fig. 6A, B). While the LB layer of VC-supplemented *Gulo*^*−/−*^ placentas exhibited a well-structured vascular network, that of VC-deficient *Gulo*^*−/−*^ placentas displayed a disheveled and strip-shaped vascular network with dilated MBSs, expanded FBV spaces, and sparse trophoblastic layers separating the MBSs from FBV (Fig. 6A, B). Immunostaining of MBSs and FBV using antibodies against cytokeratin (CK) and laminin further demonstrated an augmented density of MBV and FBV in *Gulo*^*−/−*^ placentas at E16.5 (Fig. 6C, D). The above results suggest that VC deficiency causes severe structural defects in the *Gulo*^*−/−*^ placenta, potentially contributing to the failure of maintenance of pregnancy.

Subsequently, in situ hybridization or immunohistochemistry staining was performed to detect the expression of trophoblastic markers in the *Gulo*^*−/−*^ placentas. P450scc, a maker of P-TGCs, exhibited robust expression in E14.5 placentas supplemented with VC but sporadic expression in those lacking VC (Fig. 6E). VC deficiency led to a 59% reduction in *Tpbpα*-positive areas in the junctional zone of placentas compared to VC supplementation (Fig. 6F). Likewise, the areas positive for *Plf*-, *Pl-II*-, and *Ctsq* in the labyrinth of placentas decreased significantly by 47%, 48%, and 64%, respectively, compared to VC-supplemented *Gulo*^*−/−*^ placentas (Fig. 6G–I), aligning with the in vitro findings (Fig. 1A–C) In addition, although the labyrinth expression of *SynA*, a marker of ST-I trophoblasts, seemingly remained unaffected by VC deficiency, the labyrinth expression of *SynB*, a marker of ST-II trophoblasts, notably increased with VC deficiency compared to VC supplementation (Fig. 6J, K), emphasizing compromised trophoblast functions. Finally, we examined the tri-laminar interhemal barrier (TIB) of the placental labyrinth via transmission electron microscopic. Labyrinths from VC-supplemented *Gulo*^*−/−*^ placentas showed a typical TIB structure consisting of an S-TGC layer and two tightly adhered syncytiotrophoblast layers (ST-I and ST-II) lining the fetal endothelium and separating the MBL from FBV (Fig. 6I). Conversely, labyrinths from VC-deficient *Gulo*^*−/−*^ placentas showed almost normal fetal endothelium with a contiguous basement membrane around ST-II, an atrophied S-TGC layer apparently lacking cell protrusions, an expanded ST-I layer adhering to S-TGC layer filled with dilated cytoplasmic vacuoles hindering intracellular transport, and an enlarged ST-II layer with excess lipid inclusions (Fig. 6I). Taken together, all these findings collectively suggest VC deficiency substantially activates JNK signaling to diminish Hand1 protein, facilitating developmental defects in trophoblasts within mouse placentas, particularly impacting the differentiation of multiple TGC subtypes, possibly accounting for the breakdown of maintenance of pregnancy.

## Discussion

**Fig. 7:**
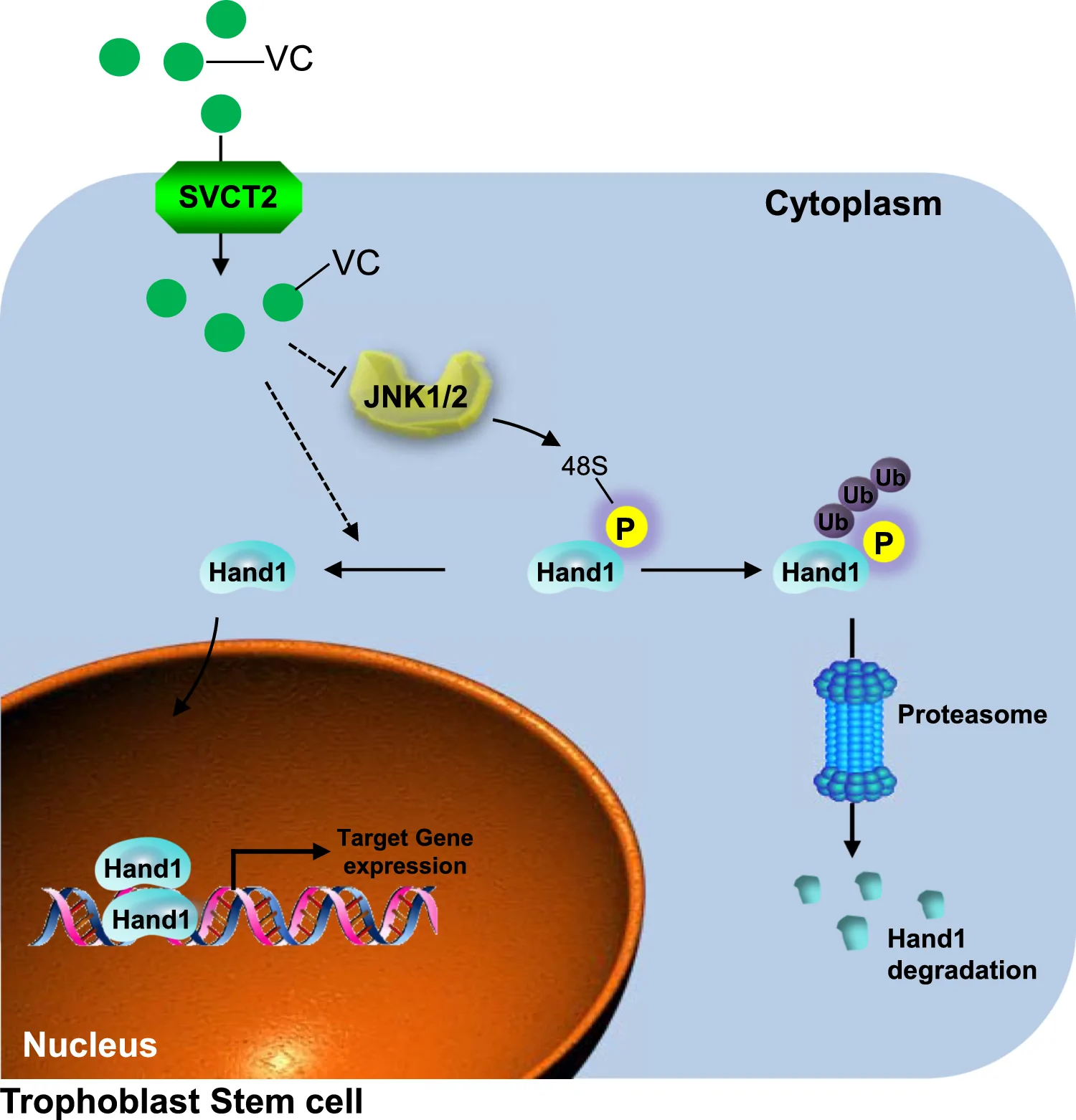
A working model for VC-mediating placentation and pregnant maintenance. VC treatment inactivates the JNK to attenuate the phosphorylation of Hand1 at Ser48 and thereby decreases the ubiquitination-dependent proteasomal degradation of Hand1, resulting in the stabilization of Hand1 and induction of trophoblast giant cell differentiation during placentation and pregnant maintenance

VC is a crucial nutrient for stem cell functions, guiding the differentiation of mesenchymal stem cells towards osteoblasts, chondrocytes, and tendons, and enhancing somatic cell reprogramming by governing epigenetic modification to generate induced pluripotent stem cells [31–34]. However, the precise roles of VC on TSCs differentiation and placentation have not been fully elucidated. By using both biochemical and genetic approaches, we have uncovered a signaling cascade that collaborates with JNK inactivation to elevate Hand1 stability following VC administration. Studies in vitro substantiate a model wherein VC likely infiltrates the cytoplasm to inactivate JNK signaling, thereby diminishing the phosphorylation of Hand1 at Ser48. This, in turn, results in Hand1 stabilization and prompts TGC differentiation throughout placentation (Fig. 7). Our findings not only corroborate earlier research highlighting the significance of MAPK signaling in TSC maintenance and differentiation [25, 35], but also pinpoint VC-induced JNK inactivation and subsequent Hand1 stabilization as an integral component in trophoblastic differentiation, placentation and potentially the sustenance of pregnancy.

Our in vivo data suggest that VC deficiency leads to severe developmental defects in TGC subtypes, including primary P-TGCs in E8.5 placentas, secondary P-TGCs, SpA-TGCs in the junctional zone, and S-TGCs in the labyrinth of E16.5 placentas. Moreover, VC treatments seemingly increase *Tpbpα* mRNA levels in the differentiation of TSCs, while VC deficiency consistently diminishes *Tpbpα* mRNA in the junctional zone of placentas. These results contrast with reports stating that Hand1 antagonizes the Ascl2 to prompt SpTs differentiation in the junctional zone [28]. The discrepancy might hint at VC’s regulation of Ascl2-mediated transcription. Besides, although VC treatment does not affect *SynA* and *SynB* mRNA levels in the differentiation of TSCs, VC deficiency notably upregulates *SynB* mRNA expression and enlarges the ST-II layer in murine placentas. The disparity could stem from insufficient S-TGCs development, prompting compensatory increments in the ST-II trophoblast layer to meet the demands of maternal–fetal exchanges for the survival of pups during VC-deficient gestation.

FGF4-mediated TSCs maintenance relies on MEKK4 activation of JNK signaling, whereas MEKK4-inactive TSCs are selectively differentiated into S-TGCs with increased expression of *Ctsq* even in the presence of FGF4 [25]. Moreover, overexpression of Hand1 in TSCs promotes TGCs differentiation even in the presence of FGF4 [36]. Besides, VC not only counteracts tumor necrosis factor-α (TNFα)-induced P38 activation in inflammatory conditions but also activates ERK1/2 to phosphorylate Runx2 to enhance osteoblastic differentiation [37–39]. Alongside our previous work demonstrating DHA’s impact on JNK activity in placental steroidogenesis regulation [26], the current study underscores the importance of JNK signaling inactivation for VC-induced TGCs differentiation.

Oxidative stress significantly impacts the maternal–fetal interface during gestation, influencing normal placentation and potentially leading to complications such as miscarriage, pre-eclampsia, and intrauterine growth restriction [40]. This notion prompts us to investigate how intracellular redox status affects MAPK activity and subsequent TGCs differentiation in response to VC stimulation. Despite the reduction of reactive oxidative species (ROS) content in TSCs with VC treatment, the disruption of intracellular redox status in TSCs does not affect basal or VC-induced TGCs differentiation. VC is unique in that it serves as a cofactor for various enzymatic reactions, including the hydroxylation of proline and lysine residues during collagen synthesis and the regulation of epigenetic modifications through the modulation of histone and DNA demethylases [33, 41–43]. These specific functions differ from the general antioxidant properties provided by other compounds such as NAC, VE, GSH, and LA, which primarily serve to neutralize ROS, indicating the role of VC in promoting TSCs differentiation operates independently of its antioxidative effects. Future investigations are needed to deeper explore the cellular differentiation mechanisms of VC-induced TSCs differentiation.

Humans possess a mutated version of the *Gulo* gene, resulting in the inability to synthesize VC, while VC deficiency impairs collagen synthesis, exacerbating the more severe symptoms of scurvy [44, 45]. In parallel, genetic ablation of *Gulo* in mice has enabled the exploration of VC’s role in various biological functions. However, investigations of *Gulo*^*−/−*^ mice demonstrate that although VC is essential for survival, its absence does not cause a measurable change in collagen production and vascular function [45]. This notion supports the speculation that VC deficiency-induced defects in placentation and pregnancy maintenance may arise from impaired trophoblastic differentiation rather than collagen synthesis.

In the absence of supplemental VC to matrices, *Gulo*^−/−^ embryos display minimal VC levels, while *Gulo*^+/-^ embryos can maintain a level of 50 μM VC. Meanwhile, maternal VC insufficiency fails to maintain the pregnancy in *Gulo*^*−/−*^ placentas and embryos, whereas *Gulo*^+/-^ placentas and embryos exhibit normal pregnancy maintenance. These findings indicate that *Gulo*^+/-^ embryos (and/or placentas) self-synthesize adequate VC to support their normal growth and development, highlighting how embryo- and placenta-specific VC scarcity leads to severe placental phenotypes and pregnancy maintenance failure. While our study underscores the importance and underlying mechanism of VC deficiency in placentation and maintenance of pregnancy, pivotal questions remain unanswered: (1) Does VC deficiency-resultant malformation of placentas indeed account for the failure of pregnancy maintenance? (2) Given the huge difference between mouse and human placentas [4, 46], does clinical VC deficiency indeed cause placental malformation, accounting for the failure of pregnancy maintenance? Further studies are needed to address these issues in the future.

While persistent deprivation of VC causes scurvy in specific conditions, certain populations encounter heightened risks of VC deficiency, such as individuals living on limited incomes, those with medical conditions like Crohn’s disease or ulcerative colitis, persons with alcohol or drug addiction, pediatric patients with advanced chronic kidney disease, older adults with restricted diets, and smokers [47–51]. Understanding that VC inactivates JNK to stabilize Hand1 in the regulation of placentation and maintenance of pregnancy carries significant clinical implications. Though VC supplementation not being widely endorsed for preventing pregnancy-associated disorders [20–23], insufficient VC uptake or deficiency could represent a crucial element accounting for the failure of pregnancy maintenance.

Numerous studies have emphasized the significance of Hand1 in facilitating the differentiation of TSCs into TGCs through the formation of Hand1 homodimers or heterodimers [5, 6, 24, 27, 28]. However, this study encountered limitations, notably the inability to conduct Hand1 knockout experiments in mouse TSCs due to technical challenges associated with cell transfection. This constraint has hindered a direct assessment of the specific role of Hand1 in TSCs differentiation. Future studies with improved transfection methods or alternative approaches may offer further insights into Hand1’s function in TSCs differentiation. Moreover, numerous studies have demonstrated that VC assists in iron absorption and metabolism [16, 52]. Given that iron is vital for brain development in fetuses, neonates, and children, and deficiencies can negatively impact myelination, neurotransmitter synthesis, and brain programming, and severe iron deficiency in neonates often results in preferential allocation of available iron to red blood cells at the expense of brain, heart, and muscle tissues [53–55]. Based on these findings, it is plausible that Fe imbalance could play a role in the regulation of TSC differentiation and pregnancy maintenance by VC. To address the potential confounding factor of iron imbalance, future experiments include measurements of iron levels and supplementation studies are needed to distinguish between the effects of VC deficiency on iron metabolism and its direct impact on trophoblast differentiation and pregnancy maintenance.

## Materials and Methods

### Animals

C57BL/6 mice were from Shanghai SLAC Laboratory Animal Co. (Shanghai, China) and ODS rats were obtained from CLEA Japan, Inc. (Tokyo, Japan). L-gulono-γ-lactone oxidase (*Gulo*^*−/−*^) global knockout mice with a C57BL/6 genetic background were generated as described previously [29, 38, 56]. *Gulo*^*−/−*^ mice received daily prepared tap water containing VC at a concentration of 4 g/L (Sigma, St. Louis, MO), while ODS rats were provided daily prepared tap water containing VC at a concentration of 1 g/L [30]. All the animals were housed in a specific pathogen-free animal facility and allowed free access to water and regular rodent chow. All animal care and handling procedures were approved by the Institutional Animal Care and Use Committee of Zhejiang University. *Gulo*^*−/−*^ females were mated with *Gulo*^*−/−*^ males to produce *Gulo*^*−/−*^ placentas and littermates, whereas *Gulo*^*−/−*^ females were mated with WT males to produce *Gulo*^+*/−*^ placentas and littermates. Female ODS rats were bred with ODS males to produce ODS placentas and littermates. The day that the vaginal plug was first observed was defined as E0.5, and placentas and littermates were harvested for analysis of phenotypes from E8.5 to E18.5.

### Blastocyst infection and transplantation

Full-length mouse Hand1 cDNA was cloned from mouse primary TSCs using the specified primers (Hand1 WT in Supplementary Table 1) with BamHI and KpnI sites. The cDNA was then inserted into pXJ-40-Flag (Flag-Hand1), pXJ-40-HA (HA-Hand1) and pXJ-40-Myc (Myc-Hand1) expression vectors (Addgene, Cambridge, MA). Site-directed mutagenesis (Stratagene, La Jolla, California) was used to introduce Hand1 mutations at Ser33, Ser48, Ser81, Ser98, Thr107, and Ser109 with specific primers (Supplementary Table 1). The mutated Hand1 variants were subcloned into pGEX-4T at BamHI and XhoI sites and the pCDH-CMV-MCS-EF1-copGFP lentiviral expression vector (System Biosciences, Palo Alto, CA) at XbaI and EcoRI sites. Lentiviral vectors expressing JNK1/2-shRNA were constructed by inserting synthesized complementary oligonucleotides (Supplementary Table 2) into the BamHI and NotI sites of pSicoR-GFP. The integrity of all constructs was verified by DNA sequencing. Lentiviruses expressing JNK1/2-shRNA or Hand1 variants were generated as described previously [3, 57], with lentiviruses having titers exceeding 1× 10^7^ CFU/ml for blastocyst infection.

Pre-implanted blastocysts at E3.5 were harvested by flushing the uterus. After removal of zona pellucidae with acidic tyrode’s solution (Sigma) as described previously [3], blastocysts were infected with lentiviruses expressing mouse Jnk1/2-shRNA or Hand1 variants for 8 h. Following observation under a stereo fluorescence microscope, blastocysts were transplanted into pseudopregnant C57BL/6 females. Typically, four to five blastocysts successfully infected with lentiviruses were transplanted into each uterine horn. Mice were sacrificed at indicated gestational stages, and various analyses were conducted on the whole placenta and fetus.

### Cell cultures, plasmids, treatments, and transfections

The TgRS26 line of mouse primary TSC (passage 2) was gifted from Professor Haibing Wang (Institute of Zoology, Chinese Academy of Sciences, Beijing, China) and cultured as described previously [58]. The proliferation medium consisted of RPMI1640 (Life Technologies, Carlsbad, CA) containing 20% fetal bovine serum (FBS, Ausbian, Australia), 2 mM L-glutamine, 1 mM sodium pyruvate, 50 μg/ml penicillin/streptomycin, 100 μM β-mercaptoethanol, 25 ng/ml FGF4 (PeproTech, Rocky Hill, NJ), 1 μg/ml heparin (Sigma) and 70% of media conditioned by primary embryonic fibroblast cells for two days. Differentiation media simply lacked FGF4, heparin and embryonic fibroblast conditioned medium. Experiments were conducted using TSCs between passages 3 and 7. TSCs were treated with specified concentrations of freshly prepared VC stock solution, anisomycin (Sigma), SB203580 (Selleck Chemicals, Huston, TX), or SP600125 (Selleck Chemicals) for 48 h, 72 h or as indicated.

Human choriocarcinoma JEG-3 cells and 293T cells were obtained from the American Type Culture Collection (Manassas, VA) and maintained as described previously [13, 57]. Transfection experiments were performed exclusively JEG-3 cell line due to its favorable transfection efficiency. The decision to utilize JEG-3 cells across these experiments was based on the potential challenges arising from the high nucleus-to-cytoplasm ratio of mouse TSCs, which could impact transfection success rates.

Constitutive active forms of JNKs (JNK*), including Flag-JNKK2-JNK1 (JNK1*) and Flag-JNKK2-JNK2 (JNK2*) were constructed through fusing JNKK2 with JNK1 or JNK2 fragments (cDNA gifted from Professor Jiahuai Han at Xiamen University, Xiamen, China) together by CloneExpress MultiS One Step Cloning Kit (Vazyme, Nanjing, China) and specified primers (Supplementary Table 2). JEG-3 or 293T cells transfected with Hand1 variants, JNK1*/2*, HA-ubiquitin (gifted from Dr. Lihui Li at Fudan University, Shanghai, China) for 6~8 h by using Lipofectamine 3000 (Life Technologies). Cells were treated with VC, anisomycin, SB203580, SP600125, or MG132 (Sigma) at 24 or 48 h post-transfection.

### Western blotting, cytosol and nuclear fractions, and co-immunoprecipitation

Western blotting was performed as described previously [57], and protein extracts were separated on 10% or 15% SDS-PAGE. The primary antibodies used as follows: p-P38, p-ERK1/2, p-JNK, t-P38, t-ERK1/2, t-JNK (Cell Signaling Technology, Danvers, MA), Hand1 (Abcam, Cambridge, UK), Flag (OriGene Technologies, Rockville, MD), Myc, HA, β-actin, glyceraldehyde-3-phosphate dehydrogenase (GAPDH), Lamin-B, IgG, GST (Santa Cruz Biotechnology, Santa Cruz, CA). Cellular cytosol and nuclear fractions were isolated using NE-PER Nuclear and Cytoplasmic Extraction Reagents (Thermo Scientific, Waltham, MA) as described previously [59]. Co-immunoprecipitation was carried out using Protein A/G Plus-Agarose Immunoprecipitation Reagent (Santa Cruz Biotechnology) with IgG, Flag or Myc antibodies, following established procedures [59]. GAPDH or β-actin served as the internal standard of total target proteins, while phosphorylated proteins were normalized to their total proteins. Immunoreactive bands from triplicates (n = 3) were semi-quantified by Image J software (ImageJ; NIH, Bethesda, MD; https://imagej.nih.gov/ij/), with the mean intensity from the first band set to 1.

### Recombinant Hand1 variants and in vitro phosphorylation assay

Recombinant glutathione-S-transferase (GST)-tagged Hand1 was generated as described previously [60], and purified utilizing Pierce™ Glutathione Superflow Agarose (ThermoFisher Scientific, Waltham, MA). An in vitro kinase assay was performed to assess Hand1 phosphorylation by JNK, as previously described [61]. To validate JNK as the kinase responsible for phosphorylating Hand1 at Ser48, recombinant active JNK2α2 (Life Technologies) and 30 ng of each GST-Hand1 variant (WT and S48A mutant) were incubated in a 50 μl of kinase assay buffer (Cell Signaling) containing γ-^32^P ATP (China Institute of Atomic Energy, Beijing, China) at 37 ºC for 30 min, followed by a 5 min boiling step. The reaction products were subjected to SDS-PAGE, and transferred to a PVDF membrane, followed by autoradiography. Subsequently, Western blotting with anti-GST was performed on the same membrane used for autoradiography.

### VC determination and quantitative RT-PCR

VC concentrations in the sera and whole embryos were determined as described previously [29]. The fresh sera and supernatants of embryo homogenates were mixed with an equal volume of cold and fresh 10% metaphosphoric acid containing 2 mM EDTA. The supernatants were frozen immediately on dry ice and kept at − 80 °C until analysis. Twenty microliters of the mixture were injected into the HPLC system with a 4.6 × 150 mm Atlantic dC18 5 μm column (Waters Co., Milford, MA). The mobile phase was methanol-phosphoric acid (80: 20, pH 2.0) at a flow rate of 0.8 ml/min, and VC elution was monitored at 254 nm by spectrophotometric analysis.

mRNA from cells was isolated using TRIzol reagent (Takara Biotechnology, Dalian, China) as per the manufacturer’s instruction. A total of 2 μg mRNA was reverse transcribed using SuperScript III Reagent (Life Technologies). mRNA levels were quantified by quantitative RT-PCR using specific primers (Supplementary Table 3), with mRNA levels of target genes normalized to those of β-actin. The relative difference in mRNA levels was calculated by 2^−ΔΔCt^ method.

### Immunostaining, in situ hybridization, and transmission electron microscopy

Immunocytochemistry was performed on chamber slides (Nalge Nunc International, Rochester, NY) as described previously [59]. Cells were immunostained with primary antibodies against Flag (OriGene Technologies) and counterstained with 4’,6-diamidino-2-phenylindole (DAPI). Immunoreactive protein signals were examined by confocal microscopy. Immunohistochemistry on placental cryosections involved antibodies against P450scc (Abcam), CK (Dako, Glostrup, Denmark) and laminin (Sigma), as described previously [3].

Digoxygenin (DIG)-11-UTP-labeled RNA probes were synthesized by in vitro reverse transcription of linearized constructs including *Pl-1*, *Tpbpa*, *Plf*, *Pl-II*, and *SynA* (all gifted from Professor Haibing Wang at Institute of Zoology, Chinese Academy of Sciences, Beijing, China), *Ctsq* and *SynB* (gifted from Dr. James C. Cross at Department of Comparative Biology and Experimental Medicine, Faculty of Veterinary Medicine, University of Calgary, Calgary, Alberta, Canada) as described previously [6, 62]. In situ hybridization was performed on placental cryosections at E16.5 as described previously [63], and detected with an alkaline phosphatase-coupled anti-digoxigenin antibody. Alkaline phosphatase activity is visualized with 5-bromo-4-chloro-3-indolyl-phosphate (BCIP) and nitro-blue-tetrazolium (NBT). The areas of positive staining for these markers in six random profiles in each placental section were quantified using Image-Pro Plus 6.0 software (Media Cybernetics, Silver Spring, MD). Histological analyses were performed in a blinded manner.

TEM examination was performed as previously described [3]. Placenta specimens were fixed with 2.5% glutaraldehyde in 0.1 M cacodylate buffer at 4 °C, post-fixed with 2% osmium tetroxide in 0.1 M cacodylate buffer, stained with 2% uranyl acetate in 0.1 M cacodylate buffer containing 30% methanol, then dehydrated and embedded in Epon812 (Electron Microscopy Sciences, Hatfield, PA). The ultrathin Sections (80 nm) were contrasted with 4% uranyl acetate and 0.25% lead citrate. Finally, the sections were examined using an FEI Technai 12 microscope (FEI Corporate, Hillsboro, OR) operating at 80 kV. TEM was carried out at the Facility Core of Microscopic Imaging, Zhejiang University School of Medicine.

### Statistical analysis

Numeral data from animals and cells are expressed as Means ± SD. Statistical analyses were performed using one-way ANOVA and Dunnett’s multiple comparison test or Student’s* t*-test (GraphPad Software Inc., La Jolla, CA). Statistical significance was assessed at *p* < 0.05 and *p* < 0.01.

## Supplementary Information

Below is the link to the electronic supplementary material.

Supplementary file1 (DOCX 22 KB)

Supplementary file2 (PDF 323 KB)

## Data Availability

All the data supporting the findings of this study are available from the corresponding author upon reasonable request.
