# Supplementary material for: Vitamin C inactivates c-Jun N-terminal kinase to stabilize heart and neural crest derivatives expressed 1 (Hand1) in regulating placentation and maintenance of pregnancy
Source: Cell Mol Life Sci. 2024 Jul 15;81(1):303. doi: 10.1007/s00018-024-05345-6 (PMC11335227; doi:10.1007/s00018-024-05345-6)
Supplement: Supplementary file 1 — Supplementary file1 (DOCX 22 KB) [file 18_2024_5345_MOESM1_ESM.docx]

**Supplemental Table 1** Primers used for construction of Hand1 wild type and mutants.

| **Gene** | **Primers (5’-3’)** |
| --- | --- |
| Hand1 WT-U | CGCGGATCCATGAACCTCGTGGGCAGCTACGCAC |
| Hand1WT-L | CGGGGTACCTCACTGGTTTAACTCCAGCGCCCAG |
| Hand1 S33A-U | GCGCGCTGTCATCAGGAAAGGCCCT |
| Hand1 S33A-L | GGCCGGACCGAAGAGGAAGGGTTCG |
| Hand1 S48A-U | GCCCCGGCTGACGCTGCCCCGGACT |
| Hand1 S48A-L | CAGCAGCCAGCTCTGGAAGTAGGGC |
| Hand1 S48D-U | GACCCGGCTGACGCTGCCCCGGACT |
| Hand1 S48D-L | CAGCAGCCAGCTCTGGAAGTAGGGC |
| Hand1 S81 A-U | GCCCCCGGGCGGCTGGAGGCGCTTG |
| Hand1 S81 A-L | CTGCCCAGGCCTGGCGTCAGGACCA |
| Hand1 S98A-U | GCAGGACCCAAGAAGGAGCGGAGAC |
| Hand1 S98A-L | GCCTTTCCGCCGGCCAAGACGGCCG |
| Hand1 T107A-U | GCTGAGAGCATTAACAGCGCATTCG |
| Hand1 T107A-L | GCGTCTCCGCTCCTTCTTGGGTCCT |
| Hand1 S109A-U | ACTGAGGCCATTAACAGCGCATTCG |
| Hand1 S109A-L | GCGTCTCCGCTCCTTCTTGGGTCCT |
| GST-Hand1-U | CGCGGATCCATGAACCTCGTGGGCAGCTACGC |
| GST-Hand1-L | CCGCTCGAGTCACTGGTTTAGCTCCAGCGCC |

**Supplemental Table 2** Primers used for seamless cloning of JNK1* and JNK2* and construction of shJNK1/2 and shJnk1/2.

| **Gene** | **Primers (5’-3’)** |
| --- | --- |
| JNKK2-U | GACGATGATAAGTCCGGATCCATGGCGGCGTCCTCCCTGGAACAG |
| JNKK2-L | TTCACCTTCACCTTCACCTTCACCTTCACCCCTGAAGAAGGGCAGGTGGGG |
| JNK1-U | GGTGAAGTTGAAGTTGAAGTTGAAGTTGAAATGAGCAGAAGCAAGCGTGACAAC |
| JNK1-L | CTTTAATAAGATCTGGTACCTCACTGCTGCACCTGTGCTAAAGG |
| JNK2-U | GGTGAAGGTGAAGGTGAAGGTGAAGGTGAAATGAGCGACAGTAAATGTGACAGT |
| JNK2-L | CTTTAATAAGATCTGGTACCTCATCGACAGCCTTCAAGGGGTCCC |
| shJNK1/2-U | GATCCGAAAGAATGTCCTACCTTCTTTCAAGAGAAGAAGGTAGGACATTCTTTTTTTTTG |
| shJNK1/2-L | AATTCAAAAAAAAAGAATGTCCTACCTTCTTCTCTTGAAAGAAGGTAGGACATTCTTTCG |
| shJnk1-U | GATCCGCTTCACTCTGCTGGAATTATTTTCAAGAGAAATAATTCCAGCAGAGTGAAGTTTTTTTTGC |
| shJnk1-L | GGCCGCAAAAAACTTCACTCTGCTGGAATTATTTCTCTTGAAAATAATTCCAGCAGAGTGAAGCG |
| shJnk2-U | GATCCGCTCAACTTTCACTGTTCTAAATTCAAGAGATTTAGAACAGTGAAAGTTGAGTTTTTTTTGC |
| shJnk2-L | GGCCGCAAAAAACTCAACTTTCACTGTTCTAAATCTCTTGAATTTAGAACAGTGAAAGTTGAGCG |

**Supplemental Table 3** Primers used for quantitative RT-PCR.

| **Genes**  **(Accession number)** | **Primers**  **(5’-3’)** | **Products**  **(bp)** |
| --- | --- | --- |
| ***β-actin***  NM_0073933.3 | S: CACGATGGAGGGGCCGGACTCATC  AS: TAAAGACCTCTATGCCAACACAGT | 256 |
| ***P450scc***  NM_019779.4 | S: GCTGGGCACTTTGGAG  AS: GGACGATTCGGTCTTTCTT | 185 |
| ***Pl-1***  NM_001205322.1 | S: GAGGCAGTCCACGAAAC  AS: ACCAAGCAGGGTAGTCA | 250 |
| ***Pl-2***  M14647.1 | S: CCAACGTGTGATTGTGGTGT  AS: TCTTCCGATGTTGTCTGGTG | 154 |
| ***Plf***  K02245.1 | S: TGAGGAATGGTCGTTGCTTT  AS: TCTCATGGGGCTTTTGTCTC | 112 |
| ***Tpbpa***  NM_009411.4 | A: CACAGCCAGTTGTTGAT  AS: CGTTGCCTACCTTGATAC | 112 |
| ***SynA***  NM_001013751.2 | S: AGCGTGACAGGCATACTTC  AS: AGGATCGTCTGGGTGGAG | 119 |
| ***SynB***  NM_173420.3 | S: GCAGCTGACACCCTCATTAAA  AS: TGAGCCTTAGGAGGCTGAGAT | 218 |
| ***Ctsq***  NM_029636.3 | S: AACTAAAGGCCCCATTGCTAC  AS: CAATCCCCATCGTCTACCC | 105 |
