# Supplementary material for: Vitamin C inactivates c-Jun N-terminal kinase to stabilize heart and neural crest derivatives expressed 1 (Hand1) in regulating placentation and maintenance of pregnancy
Source: Cell Mol Life Sci. 2024 Jul 15;81(1):303. doi: 10.1007/s00018-024-05345-6 (PMC11335227; doi:10.1007/s00018-024-05345-6)
Supplement: Supplementary file 2 — Supplementary file2 (PDF 323 KB) [file 18_2024_5345_MOESM2_ESM.pdf]

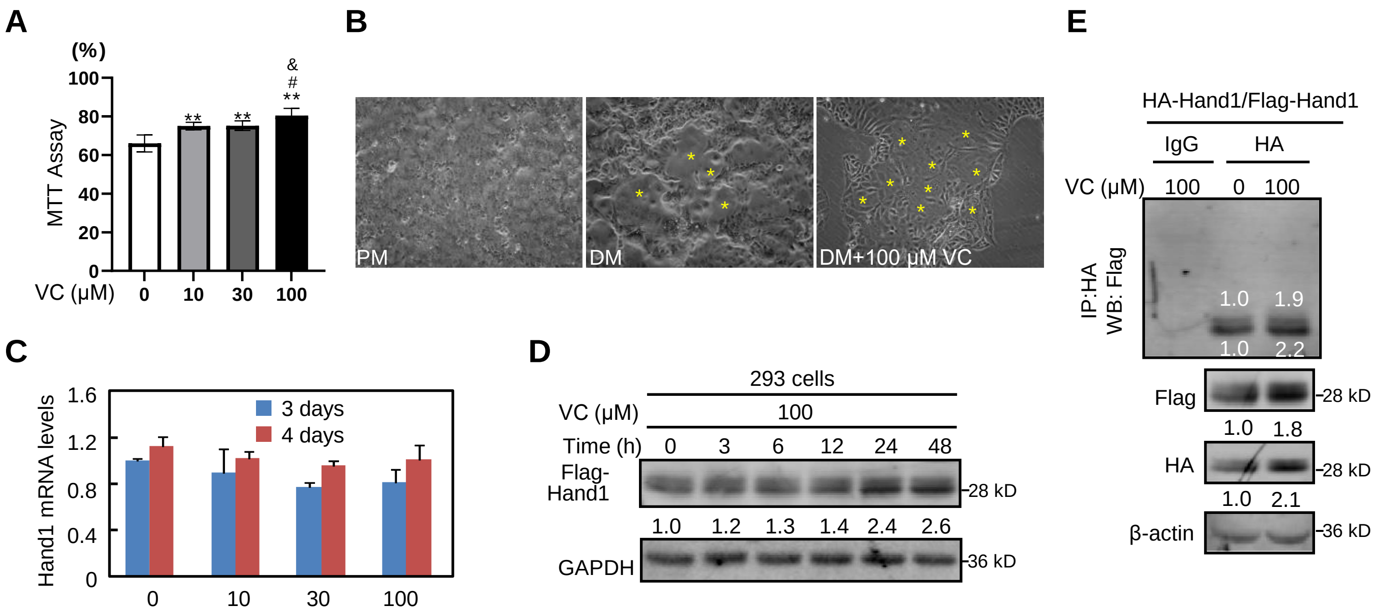

Fig. S1. VC increases exogenous Hand1 levels in 293 cells but has no effect on the *Hand1* mRNA expression in TSCs. **A** MTT assay of TSCs viability in the differentiation medium with or without VC at the indicated concentrations for 3 days. n=6. ***p*<0.01 (*vs.* 0 μM); $^{\#}$*p*<0.05 (*vs.* 10 μM); $^{\&}$*p* <0.05 (*vs.* 30 μM). **B** Bright field observation of cell morphology after culture in different mediums for 3 days, using a 10X magnification objective. PM, proliferation medium; DM, differentiation medium. The asterisks (*) highlighted in yellow denote differentiated TSC. **C** Quantitative RT-PCR assays of *Hand1* mRNA in the TSCs grown in the differentiation medium with or without VC at the indicated concentrations for 3- or 4-days. Mean ± SD, n=3. **D** Western assays of exogenous Hand1 levels in 293 cells transiently transfected with Flag-Hand1 and subsequently treated with or without VC at 100 μM for the indicated times. **E** Effects of VC on the Hand1 homodimerization. Western assays for the immunocomplex precipitated with a HA antibody from JEG-3 cells transfected with the HA-Hand1 and Flag-Hand1 and subsequently treated with or without VC at 100 μM for 24 h.

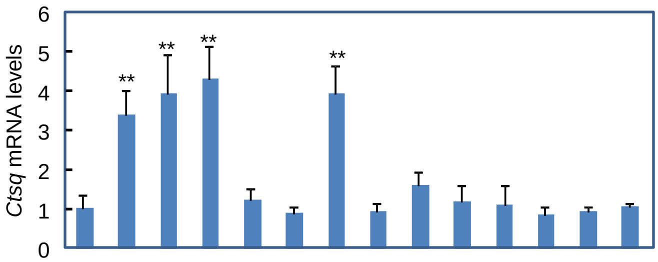

VC (μM)	-	30	100	100	-	-	100	-	-	-	-	-	-	-
H_2O_2 (μM)	-	-	-	100	30	100	-	-	-	-	-	-	-	-
DEM (μM)	-	-	-	-	-	-	100	100	-	-	-	-	-	-
LA (μM)	-	-	-	-	-	-	-	-	46.4	-	-	-	-	-
NAC (mM)	-	-	-	-	-	-	-	-	-	4	-	-	-	-
VE (μM)	-	-	-	-	-	-	-	-	-	-	100	300	-	-
GSH (mM)	-	-	-	-	-	-	-	-	-	-	-	-	1	10

Fig. S2. Effects of anti-oxidation on the expression of *Ctsq* mRNA levels. Quantitative RT-PCR assays of *Ctsq* mRNA in the TSCs grown in the differentiation medium containing the indicated concentrations of antioxidants and/or oxidants for 3 days. Mean ± SD, n=3, $^{**}p<0.01$.

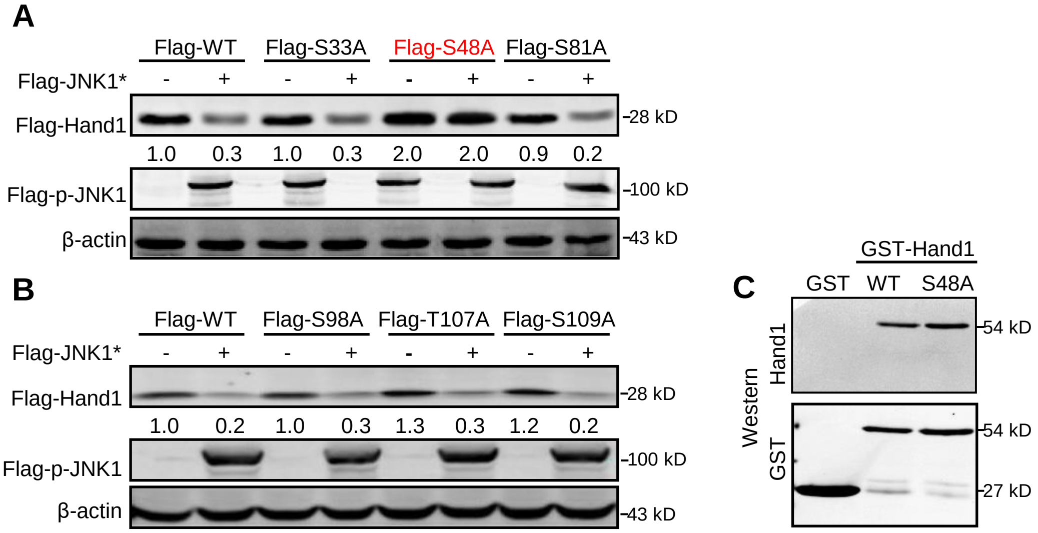

Fig. S3. Levels of Flag-Hand1 variants after JNK1 phosphorylation and identification of the recombinant GST-tagged Hand1 variants. **A-B** Western assays of Flag-Hand1 variants from JEG-3 cells, 48 hrs after transfection with Flag-Hand1 variants in combination with or without Flag-JNK1* construct. **C** Western assays of the 30 ng/lane of recombinant GST-tagged Hand1 variants by using Hand1 or GST antibody.

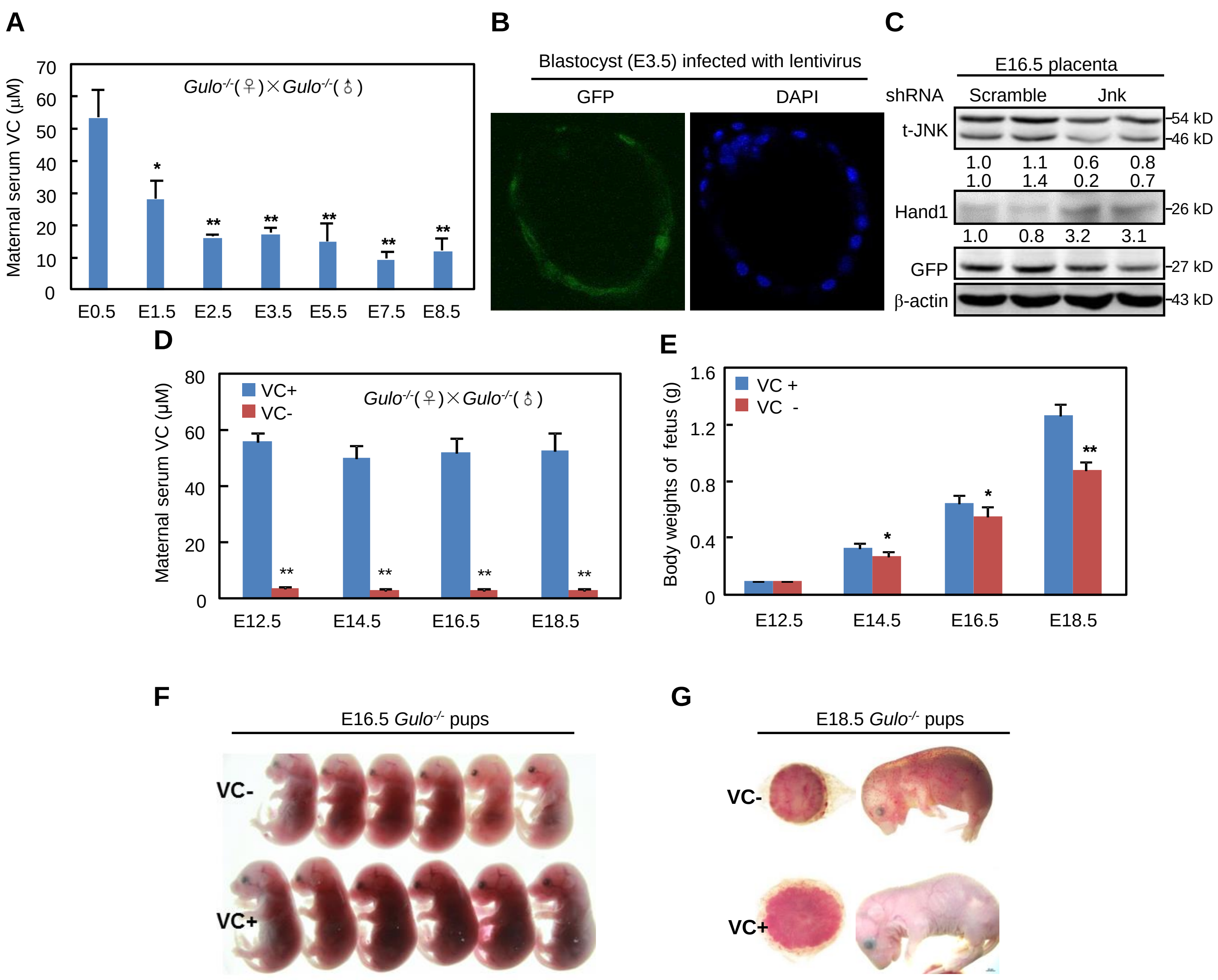

Fig. S4. VC deficiency and JNK inactivation on placentation and embryo development. **A** HPLC analyses of serum VC levels in the gestational *Gulo*$^{-/-}$ mice deprived of VC from E0.5. **B** Immunofluorescence images of GFP in the E3.5 blastocysts, 8 h after infection with GFP-bearing and Jnk1/2-shRNA-expressing lentiviruses. **C** Western assays of the JNK and Hand1 levels in the E16.5 placentas infected with GFP-bearing and scramble- or Jnk1/2-shRNA-expressing lentiviruses. **D** HPLC analyses of serum VC levels in the middle- and late-gestational stage of *Gulo*$^{-/-}$ mice supplemented with or deprived of VC at 4 g/L in the tap water from E0.5. **E-G** Body weights of embryos (**E**) and the representative images of late-gestational stage *Gulo*$^{-/-}$ embryos and placentas (**F-G**) from pregnant *Gulo*$^{-/-}$ females were assessed with or without withdrawl of 4 g/L VC in the tap water sinceplug identification (E0.5). Mean ± SD, n=6~8, *p<0.05, **p<0.01.

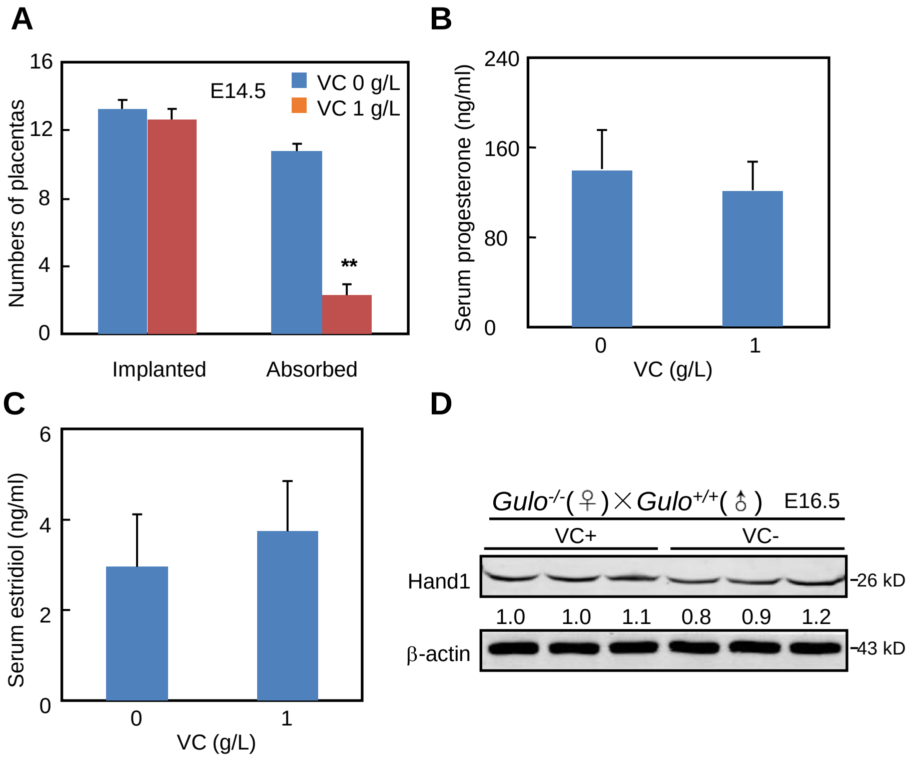

Fig. S5. Numbers of the placentas and serum P4 and E2 levels in the gestational ODS rats at E14.5 and Hand1 protein levels in placentas. **A** Numbers of implanted and absorbed placentas in the E14.5 gestational ODS rats supplemented with or deprived of VC at 1 g/L in the tap water from E0.5. **B-C** Serum levels of P4 (**B**) and E2 (**C**) in the E14.5 gestational ODS rats supplemented with or deprived of VC at 1 g/L in the tap water from E0.5. **D** Western analyses of Hand1 expression in E16.5 *Gulo*$^{+/-}$ placentas. Mean ± SD, n=6, ***p*<0.01.
